# Evaluation of a Minimally Invasive Cell Sampling Device Coupled with Assessment of Trefoil Factor 3 Expression for Diagnosing Barrett's Esophagus: A Multi-Center Case–Control Study

**DOI:** 10.1371/journal.pmed.1001780

**Published:** 2015-01-29

**Authors:** Caryn S. Ross-Innes, Irene Debiram-Beecham, Maria O'Donovan, Elaine Walker, Sibu Varghese, Pierre Lao-Sirieix, Laurence Lovat, Michael Griffin, Krish Ragunath, Rehan Haidry, Sarmed S. Sami, Philip Kaye, Marco Novelli, Babett Disep, Richard Ostler, Benoit Aigret, Bernard V. North, Pradeep Bhandari, Adam Haycock, Danielle Morris, Stephen Attwood, Anjan Dhar, Colin Rees, Matthew D. D. Rutter, Peter D. Sasieni, Rebecca C. Fitzgerald, on behalf of the BEST2 Study Group

**Affiliations:** 1 MRC Cancer Unit, Hutchison/MRC Research Centre, University of Cambridge, Cambridge, United Kingdom; 2 Department of Histopathology, Addenbrooke's Hospital, Cambridge, United Kingdom; 3 University College London Hospital, London, United Kingdom; 4 Royal Victoria Infirmary, Newcastle upon Tyne, United Kingdom; 5 Nottingham Queen's Medical Centre, Nottingham, United Kingdom; 6 Cancer Prevention Trials Unit, London, United Kingdom; 7 Queen Alexandra Hospital, Portsmouth, United Kingdom; 8 St Mark's Hospital, London, United Kingdom; 9 East and North Hertfordshire NHS Trust–QEII and Lister Hospitals, Welwyn Garden City and Stevenage, United Kingdom; 10 Northern Region Endoscopy Group, United Kingdom; 11 North Tyneside General Hospital, North Shields, United Kingdom; 12 County Durham and Darlington NHS Foundation Trust, Durham, United Kingdom; 13 South Tyneside NHS Foundation Trust, South Shields, United Kingdom; 14 North Tees and Hartlepool NHS Foundation Trust, Stockton-on-Tees, United Kingdom; McGill University, CANADA

## Abstract

**Background:**

Barrett's esophagus (BE) is a commonly undiagnosed condition that predisposes to esophageal adenocarcinoma. Routine endoscopic screening for BE is not recommended because of the burden this would impose on the health care system. The objective of this study was to determine whether a novel approach using a minimally invasive cell sampling device, the Cytosponge, coupled with immunohistochemical staining for the biomarker Trefoil Factor 3 (TFF3), could be used to identify patients who warrant endoscopy to diagnose BE.

**Methods and Findings:**

A case–control study was performed across 11 UK hospitals between July 2011 and December 2013. In total, 1,110 individuals comprising 463 controls with dyspepsia and reflux symptoms and 647 BE cases swallowed a Cytosponge prior to endoscopy. The primary outcome measures were to evaluate the safety, acceptability, and accuracy of the Cytosponge-TFF3 test compared with endoscopy and biopsy.

In all, 1,042 (93.9%) patients successfully swallowed the Cytosponge, and no serious adverse events were attributed to the device. The Cytosponge was rated favorably, using a visual analogue scale, compared with endoscopy (p < 0.001), and patients who were not sedated for endoscopy were more likely to rate the Cytosponge higher than endoscopy (Mann-Whitney test, p < 0.001). The overall sensitivity of the test was 79.9% (95% CI 76.4%–83.0%), increasing to 87.2% (95% CI 83.0%–90.6%) for patients with ≥3 cm of circumferential BE, known to confer a higher cancer risk. The sensitivity increased to 89.7% (95% CI 82.3%–94.8%) in 107 patients who swallowed the device twice during the study course. There was no loss of sensitivity in patients with dysplasia. The specificity for diagnosing BE was 92.4% (95% CI 89.5%–94.7%). The case–control design of the study means that the results are not generalizable to a primary care population. Another limitation is that the acceptability data were limited to a single measure.

**Conclusions:**

The Cytosponge-TFF3 test is safe and acceptable, and has accuracy comparable to other screening tests. This test may be a simple and inexpensive approach to identify patients with reflux symptoms who warrant endoscopy to diagnose BE.

## Editors' Summary

**Background:**

Barrett's esophagus is a condition in which the cells lining the esophagus (the tube that transports food from the mouth to the stomach) change and begin to resemble the cells lining the intestines. Although some people with Barrett's esophagus complain of burning indigestion or acid reflux from the stomach into the esophagus, many people have no symptoms or do not seek medical advice, so the condition often remains undiagnosed. Long-term acid reflux (gastroesophageal reflux disease), obesity, and being male are all risk factors for Barrett's esophagus, but the condition's exact cause is unclear. Importantly, people with Barrett's esophagus are more likely to develop esophageal cancer than people with a normal esophagus, especially if a long length (segment) of the esophagus is affected or if the esophagus contains abnormally growing “dysplastic” cells. Although esophageal cancer is rare in the general population, 1%–5% of people with Barrett's esophagus develop this type of cancer; about half of people diagnosed with esophageal cancer die within a year of diagnosis.

**Why Was This Study Done?:**

Early detection and treatment of esophageal cancer increases an affected individual's chances of survival. Thus, experts recommend that people with multiple risk factors for Barrett's esophagus undergo endoscopic screening—a procedure that uses a small camera attached to a long flexible tube to look for esophageal abnormalities. Once diagnosed, patients with Barrett's esophagus generally enter an endoscopic surveillance program so that dysplastic cells can be identified as soon as they appear and removed using endoscopic surgery or “radiofrequency ablation” to prevent cancer development. However, although endoscopic screening of everyone with reflux symptoms for Barrett's esophagus could potentially reduce deaths from esophageal cancer, such screening is not affordable for most health care systems. In this case–control study, the researchers investigate whether a cell sampling device called the Cytosponge coupled with immunohistochemical staining for Trefoil Factor 3 (TFF3, a biomarker of Barrett's esophagus) can be used to identify individuals who warrant endoscopic investigation. A case–control study compares the characteristics of patients with and without a specific disease. The Cytosponge is a small capsule-encased sponge that is attached to a string. The capsule rapidly dissolves in the stomach after being swallowed, and the sponge collects esophageal cells for TFF3 staining when it is retrieved by pulling on the string.

**What Did the Researchers Do and Find?:**

The researchers enrolled 463 individuals attending 11 UK hospitals for investigational endoscopy for dyspepsia and reflux symptoms as controls, and 647 patients with Barrett's esophagus who were attending hospital for monitoring endoscopy. Before undergoing endoscopy, the study participants swallowed a Cytosponge so that the researchers could evaluate the safety, acceptability, and accuracy of the Cytosponge-TFF3 test for the diagnosis of Barrett's esophagus compared with endoscopy. Nearly 94% of the participants swallowed the Cytosponge successfully, there were no adverse effects attributed to the device, and those participants that swallowed the device generally rated the experience as acceptable. The overall sensitivity of the Cytosponge-TFF3 test (its ability to detect true positives) was 79.9%. That is, 79.9% of the individuals with endoscopically diagnosed Barrett's esophagus were identified as having the condition using the new test. The sensitivity of the test was greater among patients who had a longer length of affected esophagus and importantly was not reduced in patients with dysplasia. Compared to endoscopy, the specificity of the Cytosponge-TFF3 test (its ability to detect true negatives) was 92.4%. That is, 92.4% of people unaffected by Barrett's esophagus were correctly identified as being unaffected.

**What Do These Findings Mean?:**

The case–control design of this study means that its results are not generalizable to a primary care population. Also, the study used only a single measure of the acceptability of the Cytosponge-TFF3 test, Nevertheless, these findings indicate that this minimally invasive test for Barrett's esophagus is safe and acceptable, and that its accuracy is similar to that of colorectal cancer and cervical cancer screening tests. The Cytosponge-TFF3 test might, therefore, provide a simple, inexpensive way to identify those patients with reflux symptoms who warrant endoscopy to diagnose Barrett's esophagus, although randomized controlled trials of the test are needed before its routine clinical implementation. Moreover, because most people with Barrett's esophagus never develop esophageal cancer, additional biomarkers ideally need to be added to the test before its routine implementation to identify those individuals who have the greatest risk of esophageal cancer, and thereby avoid overtreatment of Barrett's esophagus.

**Additional Information:**

Please access these websites via the online version of this summary at http://dx.doi.org/10.1371/journal.pmed.1001780.

- The US National Institute of Diabetes and Digestive and Kidney Diseases provides detailed information about Barrett's esophagus and gastroesophageal reflux disease
- The US National Cancer Institute provides information for patients and health professionals about esophageal cancer (in English and Spanish)
- Cancer Research UK (a non-profit organization) provides detailed information about Barrett's esophagus (including a video about having the Cytosponge test and further information about this study, the BEST2 Study) and about esophageal cancer
- The UK National Health Service Choices website has pages on the complications of gastroesophageal reflux and on esophageal cancer (including a real story)
- Heartburn Cancer Awareness Support is a non-profit organization that aims to improve public awareness and provides support for people affected by Barrett's esophagus; the organization's website explains the range of initiatives to promote education and awareness as well as highlighting personal stories of those affected by Barrett's esophagus and esophageal cancer
- The British Society of Gastroenterology has published guidelines on the diagnosis and management of Barrett's esophagus
- The UK National Institute for Health and Care Excellence has published guidelines for gastroesophageal reflux
- The Barrett's Esophagus Campaign is a UK-based non-profit organization that supports research into the condition and provides support for people affected by Barrett's esophagus; its website includes personal stories about the condition

## Abstract 3

In a multi-center case-control study, Rebecca Fitzgerald and colleagues examine whether a minimally invasive cell sampling device could be used to identify patients who warrant endoscopy to diagnose Barrett's esophagus.

## Introduction

It is estimated that 5% to 15% of adults in the Western world suffer from reflux symptoms [1], and this is the commonest physician diagnosis for gastrointestinal consultations, accounting for 9 million physician consultations in the US in 2009 [2]. On the other hand, many individuals with recurrent reflux symptoms do not consult their doctor and hence remain uninvestigated. Endoscopy is performed to justify and tailor the prescription of acid-suppressant medications, to identify early esophageal adenocarcinoma that has not yet advanced sufficiently to cause weight loss or other alarm symptoms, and to diagnose the pre-malignant precursor for esophageal adenocarcinoma called Barrett's esophagus (BE). A diagnosis of BE will be found in up to 25% of those undergoing endoscopy, depending on the severity of the symptoms and the age and sex of the individual [3–7]. The majority of individuals with BE are undiagnosed [8].

The importance of BE lies in its potential to progress to esophageal adenocarcinoma through intermediate dysplastic stages. Although the absolute risk of malignant progression is low [9,10], this is a cancer that, according to most recent data from the US Surveillance, Epidemiology, and End Results registries, has shown a more than 9-fold increase among white men, with about half succumbing to their cancer within a year. On the other hand, survival rates are high (>80%) for superficial cancers confined to the mucosa [11].

Thresholds for endoscopy referral have been the subject of vigorous debate. The major US medical societies and British Society of Gastroenterology recommend endoscopic screening for patients with multiple risk factors for esophageal adenocarcinoma, i.e., reflux symptoms, male, age above 50, high body mass index (BMI), and high waist-to-hip ratio [12,13]. Once diagnosed, patients with BE generally enter a surveillance program. Although the merits of surveillance are controversial [12,14–16], there are now strong data to support endoscopic treatment for individuals with low and high grade dysplasia, as well as intra-mucosal carcinoma, as a cancer prevention strategy [17–19].

Hence, there is a renewed interest in determining whether a more systematic diagnosis of BE coupled with endoscopic therapy could lead to lower mortality from esophageal adenocarcinoma. Endoscopic screening for all patients with reflux symptoms would not be affordable or justifiable for most health care systems. There is therefore a need for a more systematic, cost-effective, and patient-friendly diagnostic test for BE. We have developed a minimally invasive non-endoscopic cell collection device, called the Cytosponge, coupled with immunohistochemical staining for a single biomarker, Trefoil Factor 3 (TFF3), that could provide such a test. The device was piloted previously and consists of a 30-mm polyurethane sponge, contained within a capsule, which is attached to a string (S1 Fig.). The capsule is swallowed and dissolves within the stomach after 3–5 min. The sponge is then retrieved by pulling on the string, thus collecting cells on its return passage along the esophagus [20,21]. Analysis of the cell specimen for TFF3 expression provides an objective, binary read-out of the presence or absence of BE. This biomarker was ascertained from a gene expression study designed to distinguish between BE cells and those from the gastric cardia, squamous esophagus, and oropharynx, which are serially sampled by the Cytosponge as it is retrieved [21]. The current study described herein takes the previous work from a proof of concept to a case–control study powered to obtain accurate estimates of sensitivity and specificity while ensuring that the procedure remains well accepted and safe in a larger cohort of patients.

## Methods

### Study Design and Oversight

We conducted a case–control study to determine the sensitivity and specificity of the Cytosponge-TFF3 test for the detection of BE compared with endoscopy and biopsy as the reference standard (see S1 Protocol). The study was conducted according to the standards of the STARD statement for reporting studies of diagnostic accuracy (http://www.stard-statement.org/; see S1 Checklist). The UK Medicines and Healthcare Products Regulatory Agency approved use of the Cytosponge (Medical Research Council, London, UK) for this trial provided that we continued to collect safety data. Ethics approval was obtained from the East of England–Cambridge Central Research Ethics Committee (No: 10/H0308/71) and registered in the UK Clinical Research Network Study Portfolio (9461). Individual written informed consent was obtained for each patient.

### Study Participants and Setting

Consecutive patients due to attend endoscopy for a clinically indicated procedure were recruited. Cases were individuals with a previous diagnosis of BE attending for their monitoring endoscopy. BE was defined as endoscopically visible columnar-lined esophagus that measured at least 1 cm circumferentially or at least 3 cm in non-circumferential tongues (Prague classification ≥C1 or ≥M3), with documented histopathological evidence of intestinal metaplasia (IM) on at least one biopsy in the course of their endoscopic history. Controls were individuals referred to endoscopy because of dyspepsia and/or reflux symptoms. Participants who were initially enrolled as control patients but were then diagnosed with BE at endoscopy (*n* = 13) were crossed over to the case arm. Participants were recruited from across 11 centers that were geographically dispersed across the UK (S1 Table). We included four tertiary referral centers for BE (Cambridge, University College London, Newcastle, and Nottingham) to enrich for cases of BE with dysplasia, in case dysplasia adversely affected the sensitivity of the Cytosponge-TFF3 assay. Exclusion criteria were patients with bleeding diatheses or on anticoagulant medication, and those with known cirrhosis, varices, or dysphagia.

### Overview of Study Procedures

During a single office visit, we collected data on demographics, clinical exposures (alcohol, tobacco, drugs), and symptoms; administered the Cytosponge; and then performed an endoscopy procedure. Following each procedure, acceptability measures were collected. The Cytosponge was administered by a research nurse, and the whole process, including instructing the patient, took less than 10 min. Twenty-seven nurses were involved in administering the device over the course of the study across the different sites. After retrieval, the Cytosponge was placed in SurePath Preservative Fluid (TriPath Imaging, Burlington, North Carolina, US) and kept at 4°C until transportation to the laboratory.

BE patients who happened to undergo a second surveillance endoscopy for clinical purposes during the study period were invited to swallow the Cytosponge again.

### Endpoints

The primary outcomes were sensitivity and specificity of the Cytosponge-TFF3 assay for detecting BE compared with endoscopy, as well as the safety and acceptability of the device. Secondary endpoints were the sensitivity of the TFF3 screen in patients with dysplasia and in patients with BE who swallowed the Cytosponge on more than one occasion.

### Cytosponge Sample Processing

Anonymized samples were processed to paraffin blocks and cut into consecutively numbered sections. Immunostaining was carried out on slides 2 and 15 for TFF3 (proprietary monoclonal antibody; BD Diagnostics, Durham, North Carolina, US) using standard protocols on a BOND-MAX autostainer (Leica Biosystems, Newcastle upon Tyne, UK) according to GCP/GLP standards in the Tissue Bank laboratory housed within Addenbrooke's Hospital, Cambridge. One of two independent researchers (E. W. or C. S. R.) and a gastrointestinal histo-cytopathologist (M. O.) scored the slides in a binary fashion as either positive (≥1 TFF3-positive goblet cell) or negative. The expert histo-cytopathologist had the final say on TFF3 status. The scorers were unaware of the clinical diagnosis of the patient.

### Endoscopy

The gastroscopies were carried out by a local study endoscopist within an hour of the Cytosponge procedure, and patients had the choice of sedation or local anesthetic spray. A strict protocol was followed. Biopsy samples were taken from the cardia as well as 2 cm above the squamocolumnar junction in all participants, and in BE cases diagnostic biopsies were collected following the recommended Seattle surveillance protocol [22]. Diagnostic biopsies were reviewed locally. Biopsies with a diagnosis of dysplasia were reviewed in an expert consensus meeting (M. O., M. N., B. D., and P. K.). When reviewing the biopsy data, all of the histo-cytopathologists were blinded to the result of the Cytosponge test.

### Acceptability Measures

Acceptability for both the Cytosponge and endoscopy was recorded by the participant using a visual analogue scale in which 0 represented the “worst experience ever,” 10 represented the “best experience ever,” and 5 indicated “neither pleasant nor unpleasant” [20]. This acceptability measure was completed after swallowing the Cytosponge and also after the endoscopy procedure.

### Statistical Analysis

Statistical analyses were conducted by the Cancer Prevention Trials Unit (B. V. N. and P. D. S.). Assuming a sensitivity of 80% and a specificity of 90% for the TFF3 screen, we needed to recruit at least 600 BE patients and 450 controls to ensure a 95% confidence interval of approximately ±3%. Statistics for continuous variables were expressed as medians and interquartile ranges (IQRs). The Mann-Whitney test and Kruskal-Wallis test were used to compare continuous and ordinal variables, respectively, between groups, and Fisher's exact test and the Cochran-Armitage trend test [23] were used to compare counts between categorical and ordinal variables, respectively. The κ statistic for agreement between the two TFF3 scorers was calculated. Accuracy of the Cytosponge-TFF3 test was reported using Clopper-Pearson exact 95% CIs [24]. There were no missing data for the Cytosponge-TFF3 test. For the acceptability data, patients who were unable to swallow the Cytosponge and those who failed to fill in the visual analogue scale were excluded from the analysis. All reported *p*-values were two-sided. Statistical analyses were carried out using Prism V5.01, SPSS version 19, or R version 3.

### Modeling of Risk Stratification of TFF3-Positive Patients

We recently demonstrated that analysis for *TP53* mutations in Cytosponge samples can be used to diagnose high grade dysplasia (HGD) when compared with samples from patients without BE and patients with BE who had never developed dysplasia over a median follow-up of 6 y [25]. To understand the broader context of the Cytosponge-TFF3 test and stratified risk using detection of *TP53* mutations, we have modeled this in a hypothetical population of 10,000 individuals attending primary care for investigation of reflux symptoms. We used a sensitivity of 79.9% and a specificity of 92.4% for detection of any length of BE; for *TP53* mutation detection we performed the calculations using a sensitivity of 86% (95% CI 65%–96%) and a specificity of 100% (95% CI 94.6%–100%) for detection of HGD.

## Results

### Patient Characteristics and Device Safety

**Figure 1:**
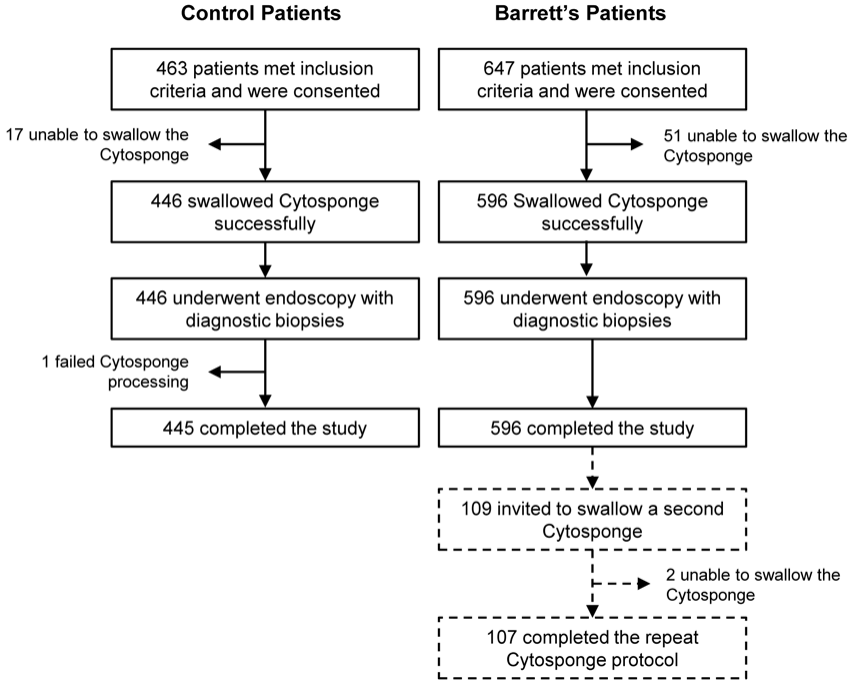
Study CONSORT diagram detailing the flow of control and Barrett's esophagus patients (cases) through the study. Patients who were unable to swallow the Cytosponge or whose Cytosponge sample failed processing were excluded from the study. Exact numbers of control and BE patients who successfully completed the study are noted.

In all, 463 controls with dyspepsia and reflux symptoms and 647 BE cases were recruited; Fig. 1 summarizes the flow of these patients through the study. The cases were generally older than the controls, with a male predominance (male:female ratio 4.0:1.0), as expected for this disease (Table 1). The median length of the BE segment for the cases was 5 cm (IQR 3–8), and 30% (176/596) of the cases had a circumferential BE segment of ≤1 cm.

**Table 1:** Demographics of the BEST2 Study patient cohorts.

Variable	Controls	BE Cases^a^
Number	463	647
Age (years)—median (IQR)	56 (44–66)	66 (58–73)
Ethnicity—number (percent)		
White	428 (92.5%)	626 (96.8%)
Other	34 (7.3%)	12 (1.8%)
Missing data	1 (0.2%)	9 (1.4%)
Male:female ratio^b^	1.0:1.3	4.0:1.0
BMI—median (IQR)	26.8 (24.0–30.2)	28.1 (25.6–31.2)
Waist-to-hip ratio—median (IQR)	0.88 (0.83–0.94)	0.95 (0.90–0.99)
Hiatus hernia—percent	34.5%	80.6%
Maximum length of BE (cm)—median (IQR)	n/a	5 (3–8)

^a^BE defined as endoscopically visible columnar-lined esophagus that measured at least 1 cm circumferentially or at least 3 cm in non-circumferential tongues (Prague classification ≥C1 or ≥M3).

^b^Sex ratio rounded to the nearest tenth.

n/a, not applicable.

Three serious adverse events were reported under the Good Clinical Practice guidelines, none of which were attributed to the Cytosponge. One patient had onset of atrial fibrillation during radiofrequency ablation that was performed at the same time as the study endoscopy. One patient was admitted for a bleed from a biopsy site, which stopped spontaneously. One patient was found to have unsuspected esophageal varices during the endoscopy, but no evidence of bleeding or Cytosponge abrasions were seen. Three days later the patient had a hematemesis, and the varices were banded. It was not possible to determine the trigger for bleeding in this case.

In terms of adverse events, 16.7% of patients had oozing of blood from a Cytosponge abrasion site (noted by the endoscopist); however, these abrasions did not require any intervention. The oozing was at worst similar to that seen at a biopsy collection site. There were anecdotal reports of a short-lived sore throat.

### Procedure Acceptability

93.9% of patients (1,042/1,110) swallowed the Cytosponge successfully. The proportion of patients who were unable to swallow the device was significantly higher in the case arm than in the control arm (7.9% versus 3.9%, *p =* 0.003). Using a fully adjusted logistic regression, increasing BMI was associated with more difficulty swallowing the Cytosponge; however, the age and gender of the patients had no impact. Of the 6% (68/1,110) of patients who were unable to swallow the device, 11.8% (8/68) were also unable to tolerate endoscopy.

**Figure 2:**
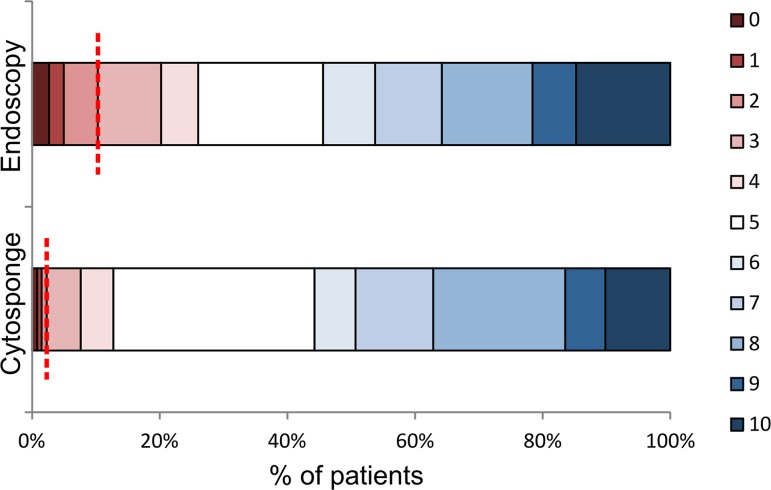
Acceptability of endoscopy and the Cytosponge test. Patients were asked to rate the procedures using a visual analogue acceptability scale after swallowing the Cytosponge and after endoscopy. The colors representing the different acceptability scores are shown on the right-hand side, with 0 representing the worst experience ever, 5 representing a neutral experience, and 10 representing the best experience ever. The dotted red line marks the boundary between mildly unpleasant or worse (left of the line, score of 0–3) and acceptable scores (right of the line, score of 4 or more), for ease of comparison between the two procedures.

When asked to rate the Cytosponge procedure using a visual analogue scale for acceptability with 0 representing the worst experience ever, 5 representing a neutral score, and 10 representing the best experience ever, 689/746 patients (92.4%) scored the experience as 4 or more, and 729/746 patients (97.7%) gave a score of 3 or more (mildly unpleasant or better), with a median value of 6 (IQR 5–8; Fig. 2). Overall, the Cytosponge procedure was rated significantly higher than the endoscopy procedure (Wilcoxon signed rank test, *p*< 0.001). In all, 258/748 patients (34.5%) rated the procedures equally, and 276/732 patients (37.8%) preferred the Cytosponge. On the whole, men rated the Cytosponge better than endoscopy (Mann-Whitney test, *p =* 0.029), and patients who were not sedated for endoscopy were more likely to rate the Cytosponge higher than endoscopy (Mann-Whitney test, *p <* 0.001).

### Diagnostic Accuracy

**Figure 3:**
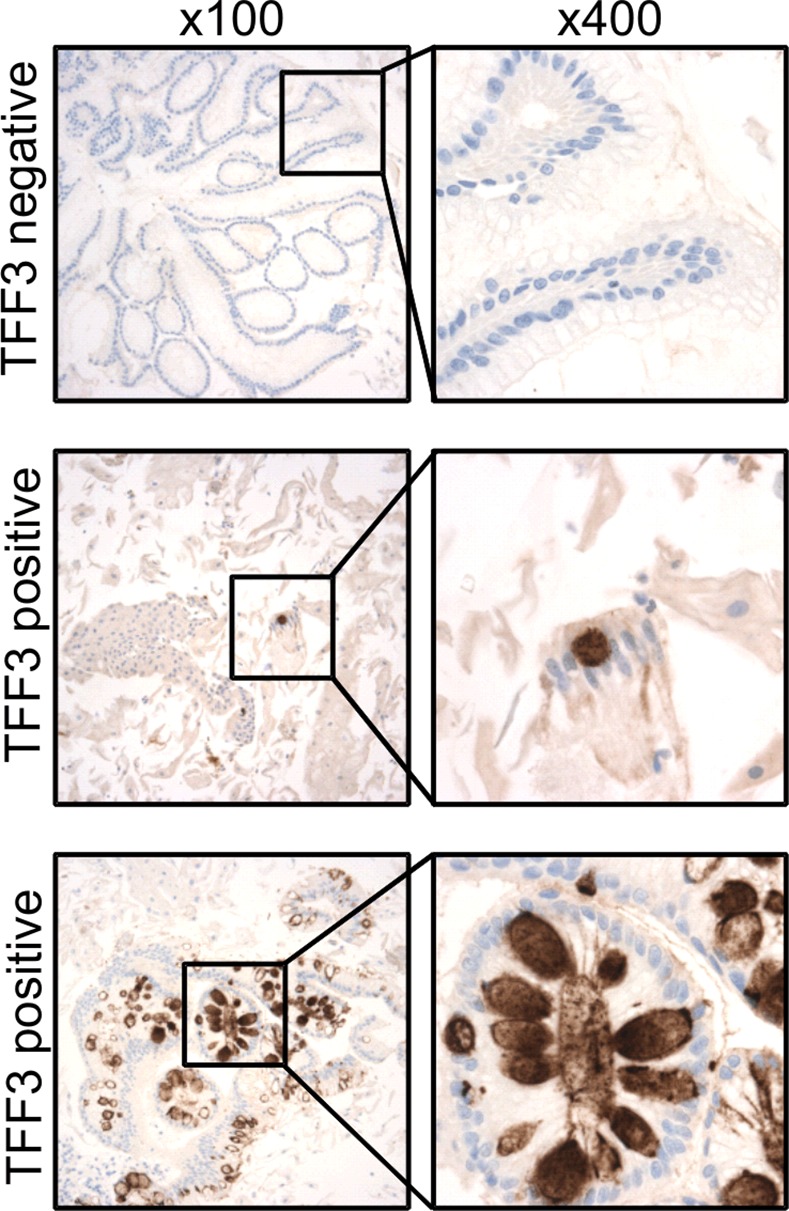
TFF3 immunohistochemical staining of Cytosponge samples. TFF3 staining was performed on all Cytosponge samples to test the sensitivity and specificity of the Cytosponge-TFF3 test for diagnosing BE. TFF3 was scored in a binary fashion, with samples with one or more TFF3-positive goblet cells being classed as positive. Shown are immunohistochemical images illustrating examples of TFF3-negative and-positive staining at low magnification (100×) and high magnification (400×).

A diagnosis of BE using the Cytosponge relies on adequate sampling of the esophageal mucosa and the presence of TFF3 positivity. Fig. 3 shows examples of TFF3 staining for a case with widespread TFF3 positivity and for a case with a single TFF3-positive goblet cell, which was also categorized as positive. A negative case is shown for comparison. The κ statistic for agreement between the two TFF3 scorers was 0.945 (95% CI 0.933–0.958), indicating substantial agreement. Overall the sensitivity and specificity for diagnosing BE according to our definition (at least 1 cm circumferentially or at least 3 cm in non-circumferential tongues, with IM in any diagnostic biopsy) were 79.9% (95% CI 76.4%–83.0%; Table 2) and 92.4% (95% CI 89.5%–94.7%), respectively (raw data available in S2 Table). There were 34 false positives for diagnosing BE (positive according to the Cytosponge-TFF3 test but negative according to endoscopy) within the 445 control patients who completed the study. None of these patients with false-positive results had IM of the gastric cardia.

**Table 2:** Sensitivity of the Cytosponge-TFF3 in different groups of patients (full dataset in S2 and S3 Tables).

Patients	Total Number	TFF3 Positive	TFF3 Negative	Sensitivity (95% CI)
All BE patients (≥C1 or ≥M3)	596	476	120	79.9% (76.4%–83.0%)
Segment length				
≥C1	533	434	109	79.5% (75.9%–82.9%)
≥C2	416	349	67	83.9% (80.0%–87.3%)
≥C3	320	279	41	87.2% (83.0%–90.6%)
Dysplasia				
NDBE	372	294	78	79.0% (74.5%–83.0%)
Indefinite for dysplasia	46	34	12	73.9% (58.9%–85.7%)
LGD	77	63	14	80.5% (69.9%–88.7%)
HGD/IMC	101	85	16	84.2% (75.6%–90.7%)
Patients having two Cytosponge tests	107	95	11	89.7% (82.3%–94.8%)

C, Circumferential length; IMC, intramucosal carcinoma; LGD, low grade dysplasia; M, maximal length; NDBE, non-dysplastic BE.

The sensitivity for diagnosing BE increased with the circumferential length of the BE segment up to 87.2% (95% CI 83.0%–90.6%, Cochran-Armitage trend test, *p <* 0.001; Table 2), and TFF3 positivity was not reduced in the presence of dysplasia (Cochran-Armitage trend test, *p =* 0.253; Table 2). Furthermore, the sensitivity and specificity were not affected by any of the clinical covariates, namely, age, gender, BMI, and waist-to-hip ratio (Table 3). A significant difference in sensitivity, but not specificity, was seen between centers (*p <* 0.001; S3 Table). This difference seems to be attributable to different proportions of cases with no columnar cells on their sample. Once cases with five or fewer columnar cell groups were excluded, the sensitivity was uniformly high (*p =* 0.24, sensitivity range 91% to 98%).

**Table 3:** Clinical covariates have no impact on sensitivity and specificity of the test.

Covariate	Sensitivity (95% CI)	Cases		Specificity (95% CI)	Controls	
		TFF3 Positive	Total		TFF3 Negative	Total
BMI (kg/m^2^)						
0–25	0.80% (0.72%–0.87%)	101	126	0.93% (0.88%–0.96%)	144	155
25–30	0.80% (0.75%–0.85%)	215	268	0.92% (0.87%–0.96%)	159	172
30+	0.80% (0.74%–0.85%)	155	194	0.92% (0.85%–0.96%)	101	110
Age (years)						
0–54.9	0.78% (0.69%–0.85%)	85	109	0.94% (0.89%–0.97%)	188	201
54.9–64.9	0.81% (0.74%–0.87%)	120	148	0.94% (0.88%–0.98%)	107	114
64.9+	0.80% (0.75%–0.84%)	271	339	0.90% (0.83%–0.95%)	116	129
Waist-to-hip ratio						
0–1	0.81% (0.77%–0.85%)	386	476	0.92% (0.89%–0.95%)	374	405
1–2	0.75% (0.65%–0.83%)	80	107	0.93% (0.78%–0.99%)	28	30
Gender						
Male	0.81% (0.77%–0.84%)	384	475	0.91% (0.86%–0.94%)	177	195
Female	0.76% (0.67%–0.83%)	92	121	0.94% (0.90%–0.97%)	234	249
Overall	0.80% (0.76%–0.83%)	476	596	0.92% (0.90%–0.95%)	411	445

The study design allowed for patients with BE to swallow a second Cytosponge if they attended for a further clinically indicated endoscopy during the time course of the study. As shown in Fig. 1, 107/109 patients (98.2%) successfully swallowed the device on a second occasion, and when both Cytosponge-TFF3 test results were considered, the sensitivity increased to 89.7% (95% CI 82.3%–94.8%; Table 2). The time interval between procedures varied depending on the clinical indication, but this did not obviously affect the result (Mann-Whitney test, *p =* 0.139).

### Modeling of Risk Stratification of TFF3-Positive Patients

**Figure 4:**
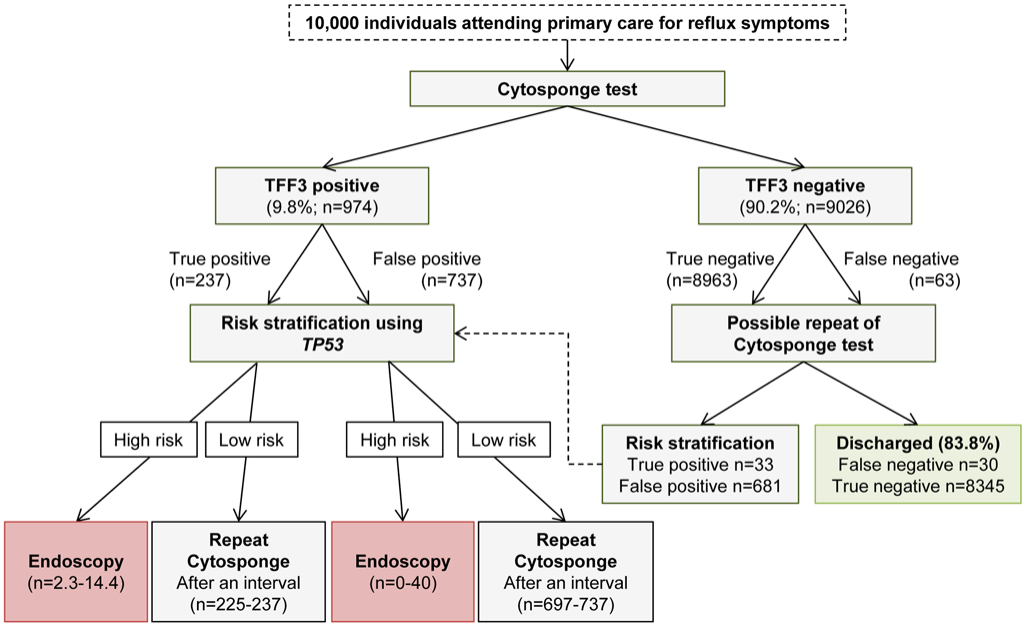
Modeling of Cytosponge-TFF3 testing and risk stratification in the primary care population with reflux symptoms. Extrapolation of findings to a hypothetical population of 10,000 individuals with reflux symptoms using a sensitivity and specificity of 79.9% and 92.4%, respectively, for the TFF3 screen, and a sensitivity and specificity of 86% (95% CI of 65%–96%) and 100% (95% CI of 94.6%–100%), respectively, for *TP53* mutation screening for detection of HGD. The assumed prevalence of BE was 3%. In patients found to be high risk, endoscopy within 6–8 wk would be recommended. For low-risk patients, a repeat Cytosponge-TFF3 test would be performed at an interval of several years (exact timing to be determined) in case they had become TP53 positive over this time period. In the TFF3-negative arm, the repeat Cytosponge testing might not be necessary. If it took place, repeat testing would be recommended within 6 to 8 wk of the delivery of the TFF3-negative results.

Risk stratification of TFF3-positive patients could be achieved through the addition of screening for further molecular markers in the same Cytosponge sample, and this strategy has the potential to significantly reduce the proportion of TFF3-positive patients who would require endoscopy. Indeed, using an exquisitely sensitive (down to an allele fraction of 0.6%) next-generation sequencing approach, we recently demonstrated that screening for *TP53* mutations in Cytosponge samples can be used to diagnose HGD [25]. We modeled risk stratification in a population of 10,000 individuals offered the Cytosponge-TFF3 test (Fig. 4). In this population, we would expect 974 (9.8%) patients to test TFF3 positive. Of these, 737 would be false positives, and following *TP53* testing, between zero and 40 patients would require endoscopy. Those testing negative for *TP53* mutations would be scheduled for a repeat Cytosponge, expected to be between 2 and 5 y later, but the optimal interval is still to be determined. Similarly, for the true TFF3-positive patients with BE (prevalence 3%), between two and 14 cases would test positive for *TP53* mutations, and approximately two of these would be confirmed dysplastic at endoscopy. Hence, following risk stratification, the endoscopy burden in the TFF3-positive arm would be significantly reduced from 974 to between two and 54 cases. On the other hand, one would expect 9,026 patients to test TFF3 negative, of which 63 would be false negatives. If sensitivity was felt to be paramount and these patients were given a second Cytosponge-TFF3 test, then one would find another 33 true positives at the expense of 681 false positives (based on our repeat Cytosponge-TFF3 test data). However, after risk stratification of these positive cases using *TP53* sequencing, again the number of endoscopies would be dramatically reduced to between two and 39 (numbers not shown in Fig. 4).

## Discussion

The Cytosponge-TFF3 test is safe and generally acceptable to patients with symptomatic reflux or dyspepsia undergoing investigation, and for those patients with BE undergoing surveillance. The test has a sensitivity of around 80% for diagnosing BE; sensitivity increases with BE segment length and is not compromised in the presence of dysplasia. The specificity of the test is 92%.

### Sensitivity of the Cytosponge-TFF3 Test

In the previous pilot study in which the primary endpoints were feasibility, safety, and acceptability, 504 patients were recruited [20]. Although not powered for accuracy, the sensitivity and specificity obtained in this initial study were 73.3% (95% CI 44.9%–92.2%) and 93.8% (95% CI 91.3%–95.8%) for ≥1 cm circumferential columnar epithelium for 15/501 patients with BE. Hence, it is very encouraging that in this adequately powered study, the overall sensitivity of the test has improved, with tighter confidence intervals (79.9%, 95% CI 76.4%–83.0%) and a similar specificity of 92.4% (95% CI 89.5%–94.7%). In both studies, the sensitivity increased with the length of the BE segment. This is highly relevant given the documented increase in cancer risk with longer BE segments [26,27]. This finding has been further confirmed by an analysis of over 1,000 cases that demonstrated an annual incidence of esophageal adenocarcinoma of 0.32% per year in patients with BE segments of 3 cm or more, compared with an annual esophageal adenocarcinoma risk of 0.04% for patients with BE segments <3 cm and 0.01% for patients with ultra-short BE segments [28].

The Cytosponge-TFF3 test sensitivity for diagnosing BE increased to nearly 90% when the device was swallowed a second time. The commonest reason for a false-negative test (76%) was the absence of columnar cells on the specimen, rather than failure of the TFF3 antibody to recognize BE when columnar cells were present. Hence, in clinical practice, it would be possible to advise patients with a TFF3-negative test and no or very few columnar cells seen on the specimen to attend for a repeat Cytosponge-TFF3 test, similar to being recalled for a repeat cervical smear because of an inadequate specimen. Alternatively, the Cytosponge-TFF3 test could be performed twice in everyone, in a manner analogous to the serial fecal occult blood testing undertaken in some countries as part of a colorectal cancer screening program. It is also possible that the device could be engineered to maximize collection of cells at the gastroesophageal junction.

### False Positives

False positives are also an important consideration for any screening or diagnostic test. Analysis of our Cytosponge-TFF3 test data in which there were 34/445 (7.6%) false positives suggests that these were not due to IM at the gastric cardia, nor were they due to very short tongues of BE. Patients with BE tongues of 1 cm or less with no IM present on biopsies were included in the control cohort as these patients are likely to have irregular Z lines, but there was no correlation between these types of patients with very short Barrett tongues and false-positive TFF3 results (*p =* 1.0). However, the correlation between false-positive TFF3 results and the presence of *Helicobacter pylori* nearly reached significance, with a *p*-value of 0.0825. *H*. *pylori* infection may lead to the presence of IM within the stomach, which may have been missed on the gastric cardia biopsy used to assess the presence of cardia IM, but which may nevertheless have been sampled by the Cytosponge. It is also known that up to 10% of patients presenting to endoscopy for dyspepsia have a diagnosis of gastric IM, a known precursor lesion to gastric cancer [29]. If a portion of these false-positive results are indeed due to gastric IM, it may actually be beneficial to the patients to be made aware of this, especially since an estimated 10% of these patients will develop gastric cancer. To put these data in context, our false-positive rate of 7.6% compares with the false positive rate of 2%–9% for colorectal screening. The new multi-target stool DNA test for colorectal cancer screening [30] has an even higher false-positive rate of 10.2%–13.4%, compared with that of the current fecal immunochemical test, which is 3.6%–5%, although the authors of the multi-target stool DNA test article argue that sensitivity is more important than specificity. Another relevant comparison is cervical cancer screening, which has a false-positive rate of 6%–15% [31], where the over-diagnosis of micro-invasive cancer is considered more than compensated by the prevention of cancer through treatment of cervical intra-epithelial neoplasia. With the advent of outpatient-based endoscopic therapy for BE, a similar argument can be applied to BE. Risk stratification could lead to the equivalent grading as for cervical intra-epithelial neoplasia 1–3.

### Acceptability and Implementation of the Test

The acceptability data are very encouraging. The same scale was used in the community-based pilot study [20], in which the median score was 7 (IQR 5–8) compared with the median of 6 (IQR 5–8) described here. It is likely that the clinical setting may alter patient preferences. The community setting is usually more familiar and less daunting for patients, and in this study the individuals were about to have an endoscopy procedure a few minutes later, which would have influenced their experience.

When considering implementation of a new technology, the logistical considerations are also important. The Cytosponge procedure was administered in 11 centers by a total of 27 nurses; training occurred via one training visit by the nurse coordinator (I. D.). In terms of sample logistics, the samples were stored at 4°C and could be kept for several weeks prior to processing. The sample processing and antibody staining were performed in a laboratory housed within the hospital pathology department, and as such followed clinical laboratory standards. Because of the binary nature of the TFF3 scoring, this test would be amenable to automated image scanning. Overall, this study was conducted using methods relevant to routine clinical practice, and nothing emerged that would seem to preclude the implementation of such a diagnostic test.

The Cytosponge-TFF3 test could be used as a first-line diagnostic test in patients with multiple risk factors for BE (as recommended by the major medical society guidelines [13,32,33]) who would otherwise be referred for endoscopy to rule out BE. However, since a previous modeling study suggested that the Cytosponge-TFF3 test combined with endotherapy for treating early cancer had cost advantages over endoscopy [34], this approach may open up the possibility for more systematic diagnosis of a broader subgroup of the population in the future. Indeed, since only 12% of patients with gastroesophageal reflux disease are currently being endoscoped, it follows that only a small fraction of BE cases are being diagnosed through the current referral routes. While the sensitivity of the Cytosponge-TFF3 test is not perfect at 80% to 90%, depending on length of BE segment and repeat Cytosponge testing, it would allow a diagnosis in the vast majority of the currently undiagnosed pool of patients, thus enabling the required step-change to improve mortality of esophageal adenocarcinoma. However, our modeling is limited to the expected number of BE patients diagnosed and does not estimate the expected number of patients who would progress to esophageal adenocarcinoma. Further modeling would be required to obtain this degree of information. Based on a 5% prevalence of BE and sensitivity of 80%, the modeled negative and positive predictive values of the test would be ~98.9% and 34.7%, respectively. However, the positive predictive value depends heavily on disease prevalence and would decrease to 25.1% for a population with a 3% BE prevalence and increase to 44.9% for patient populations with more severe reflux symptoms. Hence, the population at risk could be tailored further using simple symptoms nomograms [35,36]. These modeled values are favorable compared with current screening tests such as fecal occult blood testing, cervical smears, and mammography [31,37–38].

While the case–control design was appropriate to obtain accuracy data with narrow confidence intervals, this design has known limitations in terms of the ability to generalize the data to the primary care population consulting for reflux symptoms. Care must be taken not to extrapolate the negative and positive predictive values directly. In addition, the acceptability data were limited to a single measure. In the future it will be important to collect more in-depth qualitative data on acceptability.

### Conclusions

The Cytosponge-TFF3 test can diagnose BE in a manner that is acceptable to patients and logistically feasible across multiple centers. This test may substantially lower the threshold for investigating patients with reflux, as part of a strategy to reduce population mortality from esophageal adenocarcinoma. Analysis of a full panel of risk-stratification biomarkers enriched further for dysplasia and applied to this cohort is ongoing, and a randomized controlled trial of the Cytosponge-TFF3 test compared with the current standard of care for patients with reflux and dyspepsia symptoms in primary care is in preparation. The data from such studies will be required prior to implementation of the Cytosponge-TFF3 test on a population level.

## Supporting Information

S1 Checklist STARD checklist for reporting of studies of diagnostic accuracy (version January 2003). (DOC)

S1 Fig Cytosponge in the capsule before ingestion, as well as after the capsule has dissolved and the Cytosponge has been released. (PPTX)

S1 Protocol Up-to-date study protocol. (PDF)

S1 Table Numbers of controls and cases recruited by each center. (DOCX)

S2 Table Raw data for sensitivity and specificity analysis. (XLSX)

S3 Table Sensitivity and specificity by center. (DOCX)

## Abbreviations:

BE: Barrett's esophagus
BMI: body mass index
HGD: high grade dysplasia
IM: intestinal metaplasia
IQR: interquartile range
