# Supplementary material for: Evaluation of a Minimally Invasive Cell Sampling Device Coupled with Assessment of Trefoil Factor 3 Expression for Diagnosing Barrett's Esophagus: A Multi-Center Case–Control Study
Source: PLoS Med. 2015 Jan 29;12(1):e1001780. doi: 10.1371/journal.pmed.1001780 (PMC4310596; doi:10.1371/journal.pmed.1001780)
Supplement: S1 Fig — (PPTX) [file pmed.1001780.s002.pptx]

## Slide 1
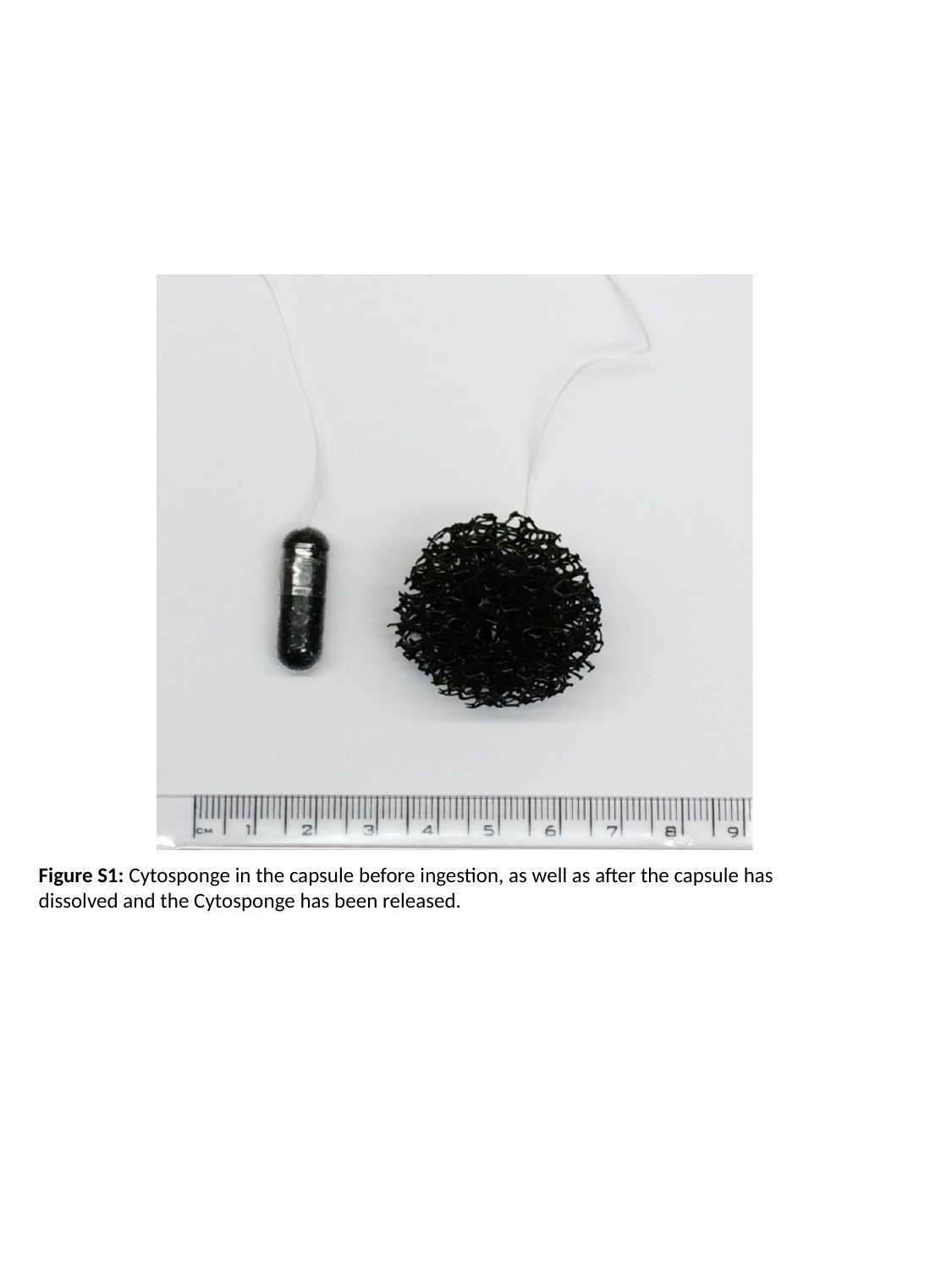

Figure S1: Cytosponge in the capsule before ingestion, as well as after the capsule has dissolved and the Cytosponge has been released.
