# Supplementary material for: Evaluation of a Minimally Invasive Cell Sampling Device Coupled with Assessment of Trefoil Factor 3 Expression for Diagnosing Barrett's Esophagus: A Multi-Center Case–Control Study
Source: PLoS Med. 2015 Jan 29;12(1):e1001780. doi: 10.1371/journal.pmed.1001780 (PMC4310596; doi:10.1371/journal.pmed.1001780)
Supplement: S1 Protocol — (PDF) [file pmed.1001780.s003.pdf]

Protocol

Evaluation of a Non-Endoscopic Immunocytological Device (Cytosponge) for Barrett's Esophagus Screening in a Case-Control Study (BEST2)

Chief Investigator: Prof Rebecca Fitzgerald

Signature: ______________________

Joint Sponsors University of Cambridge

____

Signature:

Joint Sponsors Cambridge University Hospitals NHS Foundation Trust

Signature: ______________________

CONTACTS

Joint Sponsors
University of Cambridge:
Helen Atkinson, Assistant Director, Clinical Medicine, Cambridge Research Office, University of Cambridge School of Clinical Medicine, Addenbrooke's Hospital, Box 111, Hills Road, Cambridge CB2 0SP.
Cambridge University Hospitals NHS Foundation Trust:
Stephen Kelleher. Box 277, R&D Dept, Hills Road, Cambridge University Hospitals NHS Foundation Trust, Cambridge, CB2 0QQ, Tel: 01223 217418.

Chief Investigator (CI)
Prof Rebecca Fitzgerald, Programme Leader, MRC Cancer Cell Unit, Hutchison-MRC Research Centre; Adjunct Faculty, CRUK Cambridge Research Institute and Hon. Consultant Gastroenterologist, Addenbrooke's Hospital, Cambridge.

Clinical Trials Unit
Prof Peter Sasieni, Director of the Cancer Prevention Trials Unit, Cancer Prevention Trials Unit, Wolfson Institute of Preventive Medicine, Barts and The London School of Medicine and Dentistry, Queen Mary, University of London, Charterhouse Square, London EC1M 6BQ, Tel: 0207 882 3544.

The CTU will be responsible for the trial design, statistical design and analysis, IT (database build, remote data capture etc).

Trial Co-ordinator
Mrs. Irene Debiram-Beecham, University Department of Oncology, Hutchison/MRC Research Centre, Hills Road, Cambridge CB2 0XZ, Tel: 01223 761085 / 01223 763994.

Study Statistician
Prof Peter Sasieni, as above

Trial Management Group (TMG)
CI: Prof Rebecca Fitzgerald, as above.
Principal Investigators (PIs):
Dr Laurence Lovat, Senior Lecturer and Hon. Consultant, University College London
Dr Krish Ragunath, Consultant Gastroenterologist, Associate Professor and Reader, Nottingham Queens Medical Centre
Prof. Michael Griffin, Consultant Surgeon, Royal Victoria Infirmary Newcastle upon Tyne Study Pathologist: Dr Maria O'Donovan: Consultant Histopathologist and Research Associate Addenbrooke's Hospital and MRC Cancer Cell Unit, Hutchison-MRC Research Centre, Cambridge
CPTU Director: Prof Peter Sasieni,
Trial Scientist: Dr Pierre Lao-Sirieix
Trial Coordinator: Mrs. Irene Debiram-Beecham

Participating Sites

- Addenbrooke's Centre for Clinical investigation, Addenbrooke's Hospital, Cambridge University Hospitals NHS Foundation Trust, Hills Road Cambridge, CB2 0QQ – Prof Rebecca Fitzgerald
- University College London Hospital, 235 Euston Road, London, NW1 2BU- Dr Laurence Lovat
- Nottingham Queen's Medical Centre, Derby Rd, Nottingham NG7 2UH - Dr Krish Ragunath
- Newcastle, Royal Victoria Infirmary, Queen Victoria Road, Newcastle NE1 4LP– Prof Michael Griffin
- East and North Hertfordshire Trust, Queen Elizabeth II Hospital, Howlands, Welwyn Garden City, Herts AL7 4HQ and The Lister Hospital, Coreys Mill Lane, Stevenage, Hertfordshire SG1 4AB - Dr Danielle Morris and Dr Ian Sargeant
- St Marks Hospital , Middlesex, London - Dr Adam Haycock
- Queen Alexandra Hospital, Southwick Hill Road, Cosham, Portsmouth PO6 3LY- Prof Pradeep Bhandari

North Region Endoscopy Group

- Bishop Auckland General Hospital, Cockton Hill Road, Bishop Auckland, Co Durham DL14 6AD as part of County Durham and Darlington NHS Foundation Trust, Cockton Hill Road, Co Durham, DL14 6AD - Dr Anjan Dhar
- North Tyneside General Hospital, Rake Lane, North Shields NE29 8NH- Dr Stephen Attwood
- North Tees and Hartlepool NHS Foundation Trust, University Hospital of North Tees, Stockton-on-Tees, Cleveland TS19 8PE – Dr Matt Rutter
- South Tyneside NHS Foundation Trust, South Tyneside district hospital, Harton Lane, South Shields, Tyne and Wear NE34 0PL – Prof Colin Rees

Pathology and Laboratory team

Dr Pierre Lao-Sirieix, Senior Laboratory Scientist, MRC Cancer Cell Unit, Hutchison-MRC Research Centre, Cambridge.

Dr Caryn Ross-Innes, Post Doc Research Associate, MRC Cancer Cell Unit, Hutchison-MRC Research Centre, Cambridge.

Dr Sibu Varghese, Clinical Research Fellow, MRC Cancer Cell Unit, Hutchison-MRC Research Centre, Cambridge.

Dr Maria O'Donovan, Consultant Histopathologist and Research Associate, Addenbrooke's Hospital and MRC Cancer Cell Unit, Hutchison-MRC Research Centre, Cambridge.

Professor Marco Novelli, Professor of Pathology, University College London.

Dr Philip Kaye, Consultant Histopathologist, Queen's Medical Centre, University of Nottingham.

Prof. Alastair Burt and Dr. Babette Disep, Consultant Pathologists, Royal Victoria Infirmary, Newcastle Upon Tyne

External collaborators advising on laboratory assays

Prof. Steven Meltzer, Professor of Gastroenterology, John Hopkins, Baltimore, USA (collaborator methylation biomarkers).

Dr Sheila Krishnadath, Consultant gastroenterologist, Academic Medical Center, Amsterdam, The Netherlands (collaborator FISH markers).

Independent Statistical Advisor

Dr Paul Pharoah, Senior Clinical Research Fellow, Cancer Research UK Department of Oncology, Strangeways Laboratories for Disease Prevention, Cambridge.

Advisory Committee

Mr Richard Hardwick, Representative of AUGIS & Chair National Oesophago-Gastric Cancer Audit, Oesophago-Gastric Centre, Addenbrooke's Hospital, Cambridge.

Prof Liam Murray, Professor of Cancer Epidemiology with the Centre for Clinical and Population Science in Queen's University Belfast.

Prof J Emery, Professor of General Practice, University of Western Australia and Honorary Senior Research Fellow, General Practice & Primary Care Research Unit, Dept of Public Health & Primary Care, University of Cambridge.

Prof. Jane Blazeby, Professor of Surgery and Researcher Dept. of Social Medicine, University Bristol

Dr Edwin Swarbrick, Endoscopy Vice-President, British Society of Gastroenterology, Wolverhampton NHS Trust.

Mrs Mimi McCord, Lay representative Heartburn Cancer Awareness Support.

SUMMARY / SYNOPSIS

**Title**	BEST2: Evaluation of a Non-Endoscopic Immunocytological Device (Cytosponge) for Barrett's Esophagus Screening in a Case-Control Study
**Methodology**	A case: control study design in which the cases will be patients with known Barrett's Oesophagus (BE) and controls individuals with reflux or indigestion (dyspepsia) symptoms referred for endoscopy. Four centres with expertise in Barrett's oesophagus will recruit patients. All participants will swallow the Cytosponge device prior to having an endoscopy. The Cytosponge will be processed for a number of different biomarkers. The results will be compared with the endoscopy findings.
**Study Duration**	3 years
**Objectives (summary)**	1. Safety and performance characteristics of the Cytosponge test for diagnosing BE compared with endoscopy, including specificity (from controls) and sensitivity (from cases)<br>2. Differential sensitivity of screening BE with dysplasia (low and high grade) compared to non-dysplastic BE.<br>3. Determine the reproducibility of the Cytosponge result by repeat testing in a subset of controls and Barrett's patients attending for clinically indicated repeat surveillance during the trial period.<br>*4.* For patients with BE, the ability of Cytosponge biomarkers to risk stratify patients in comparison with dysplasia grade obtained from endoscopic biopsies.<br>5. Logistics of high-throughput sample processing and automated analysis of Cytosponge specimens for use in routine NHS or other health care settings.
**Number of Participants**	500-700 cases and 500-700 controls (a range is given because this will vary slightly depending on the prevalence of dysplastic cases in order to give us 100 cases of low grade dysplasia (LGD) and 100 high grade dysplasia (HGD)).
**Main inclusion criteria**	Any patient clinically fit for an endoscopy with Barrett's oesophagus (for the cases) and (or) with upper GI symptoms of reflux or dyspepsia as an indication for endoscopy. Individuals must be able to provide informed consent.
**Statistical methodology and analysis**	Statistical methods for proportions including estimation of proportions with confidence intervals and testing for difference between two proportions and trends in proportions.

ABBREVIATIONS

Adenocarcinoma: AC
Barrett's Oesophagus: BE
BEST2 Web-Based application: BEST2 app
Case report Form: CRF
Deoxyribonucleic acid: DNA
Ethylenediaminetetraacetic acid: EDTA
Fluorescence *in situ* hybridization: FISH
Gastric Cardia: GC
Gastrointestinal: GI
Gastro-oesophageal Junction: GOJ
Good Clinical Practice: GCP
Good Laboratory Practice: GLP
High Grade dysplasia : HGD
Low Grade Dysplasia: LGD
Minichromosome maintenance protein 2: mcm2
Normal Squamous epithelium: NE
Polymerase Chain Reaction: PCR
Principal Investigator: PI
Standard Operating Procedures: SOPs
Squamous Columnar Junction: SCJ
Trefoil Factor: TFF3
Tumour protein 53: TP53

GLOSSARY

Ablation: removal of Barrett's epithelium using an endoscopy based procedure such as radiofrequency ablation or laser.

Biopsy: the removal of a small piece of tissue which in this context is done at the time of endoscopy.

Barrett's oesophagus: the replacement of the normal oesophageal lining with cells which resemble the intestine when examined under the microscope. This is sometimes referred to as intestinal metaplasia or IM.

Cyclin A: control the progression of cells through the cell cycle S phase by activating cyclin-dependent kinase (Cdk) enzymes.

Dysplasia: abnormal arrangement of cells which can be diagnosed by the pathologist when examining the biopsy. This is an intermediate step prior to cancer.

Dyspepsia: this term is interchangeable with indigestion. It refers to pain or discomfort just below the breast bone.

Dysplasia status: the grade of dysplasia. Pathologists use set criteria and grade it as low or high grade. High grade is nearer to cancer.

Endoscopy: the video camera used to examine the gastrointestinal tract. The endoscope used for Barrett's can examine the oesophagus, stomach and first part of the small intestine called the duodenum.

Endoscopic brushing: a superficial sample of cells from the oesophagus can be obtained by rubbing a small brush over the surface.

Endoscopic Mucosal resection: the removal of a larger piece of oesophagus through the endoscope. This is often used to determine how deep a cancer penetrates into the wall of the oesophagus. It can also be used for treatment since removing it can cure the problem if superficial.

FISH (fluorescence in situ hybridization) is a cytogenetic technique developed by Christoph Lengauer that is used to detect and localize the presence or absence of specific DNA sequences on chromosomes. FISH uses fluorescent probes that bind to only those parts of the chromosome with which they show a high degree of sequence similarity. Fluorescence microscopy can be used to find out where the fluorescent probe bound to the chromosomes. FISH is often used for finding specific features in DNA for use in genetic counselling, medicine, and species identification.

Heartburn: a burning sensation behind the breastbone in the chest which can rise to the back of the mouth. It has nothing to do with the heart.

High grade dysplasia: abnormal arrangement of cells which can be diagnosed by the pathologist when examining the biopsy. This is the step before cancer development.

Low grade dysplasia: slightly abnormal arrangement of cells which can be diagnosed by the pathologist when examining the biopsy.

Reflux : the movement of acid and bile from the stomach into the oesophagus and sometimes into the mouth.

Reflux symptoms: any symptoms caused by acid and bile moving into the oesophagus. Also described as heartburn and dyspepsia.

Snap Frozen: biopsies taken from tissue is immediately placed into liquid nitrogen using a cryotube.

Tertiary referral centre: a hospital providing a specialist service to which other hospitals refer e.g. for assessment and treatment of high grade dysplasia in Barrett's oesophagus.

TP53 : P53 (also known as protein 53 or tumor protein 53), is a tumor suppressor protein that in humans regulates the cell cycle and, thus, functions as a tumor suppressor that is involved in preventing cancer.

TABLE OF CONTENTS

	Protocol Headings	Location
1.	Background – Oesophageal adenocarcinoma and Barrett's Oesophagus	9-12
2.	Study objectives	12
3.	Study design	12-20
4.	Data Collection	21
5.	Selection and withdrawal of participants	23
6.	Assessment of Efficacy	25
7.	Assessment of Safety	25
8.	Statistics	27
9.	Termination of the study	29
10.	Quality Control and Quality Assurance	29
11.	Regulatory Authorities	30
12.	Ethics	30
13.	Monitoring	30
14.	Access to Source Data	30
15.	Data handling and record keeping	30
16.	Financing and Insurance	30
17.	Remit of Trial Management and Advisory Groups	31
18.	Trial Advisory Group	31
19.	Publication Policy	31
20	References	32-33

1. **Background**

Oesophageal adenocarcinoma and Barrett's oesophagus

Cancer of the oesophagus (gullet) occurs as two main types, squamous cell cancer and adenocarcinoma (AC). Adenocarcinoma has increased rapidly in the western world over the past twenty years and is now the commonest form that we see in the United Kingdom (UK) (1).Most patients present at an advanced stage of their disease and as a result the overall 5 year survival remains less than 14% (2). For these reasons, both Cancer Research UK (CRUK) and the Chief Medical Officer have highlighted this disease as a strategic priority and recommended that research should be supported to explore minimally invasive screening tests (3). The main risk factor for this cancer is the reflux of acid and bile into the oesophagus which has occurred over many years. This reflux is commonly experienced by patients as heartburn and indigestion (dyspepsia). The progression to cancer is gradual and occurs via an intermediate stage called Barrett's oesophagus (BE). BE is the replacement of the normal (squamous) lining of the lower oesophagus with a glandular lining which more closely resembles the intestine. It is diagnosed through endoscopy and biopsy. Most cases of Barrett's oesophagus are undiagnosed in the population since it is not routine clinical practice to endoscope everyone with reflux symptoms. However, if all cases could be detected then this might open up the possibility to prevent the progression of this condition to cancer. Detection of Barrett's oesophagus could be achieved by a screening programme. Hitherto the utility of screening for BE has been questionable given the lack of treatment options. However, there has been rapid advancement in technologies such as endoscopic mucosal resection (removal of an early cancer through the endoscope) and methods to "burn" away the lining of the oesophagus through techniques such as radiofrequency ablation (4-5). In addition, drug prevention measures (chemoprevention) are being evaluated in a large CRUK funded trial Aspirin Esomeprazole Chemoprevention Trial (AspECT). Therefore screening-detected cases of BE could potentially be coupled to interventions to prevent cancer thus avoiding the need for invasive treatments for established cancer which include chemotherapy and surgery to remove the oesophagus (oesophagectomy) which has significant mortality and morbidity (2).

Any screening test needs to be simple, safe, precise, validated and acceptable to the population (6). The current gold-standard endoscopic diagnosis is invasive, technical and expensive. Development of new endoscopes such as the ultrathin endoscope which can pass through the nose without sedation (ultrathin transnasal endoscopy) may improve acceptability; however it remains an invasive, expensive test requiring technical expertise (7-8). A small pill-cam (wireless video capsule endoscopy) has a high sensitivity (9); but is also high-tech, expensive and does not permit tissue sampling. Previous attempts to develop non-endoscopic screening tests which remove surface cells (cytology) using balloon like devices have failed (10).

Development of the Cytosponge Test and Pilot Data

We have developed a non-endoscopic device called a Cytosponge which previously received a letter of no objection from the Medical Healthcare products Regulatory Agency (MHRA Reference CI/2007/0053) to screen for BE (Figure 1A). It is a less invasive procedure than endoscopy and consists of an expandable, spherical mesh which is attached to a string and contained within a soluble capsule. 3-5 minutes after swallowing (once the capsule has dissolved), the spherical mesh, which measures around 3cm in diameter can be retrieved by pulling on the string. Cells from the

oesophagus are removed by the mesh scraping against the surface as it is retrieved. The sponge sample is then placed into a preservative fluid and the specimen is processed for molecular indicators of BE (biomarker evaluation), (Figure 1B). In a study comparing persons with known BE compared to healthy controls, the most promising BE biomarkers were an antibody raised against the proliferation marker minichromosome maintenance protein 2 (Mcm2); and a mucin characteristic of the BE lining (the intestinal metaplastic phenotype) Trefoil Factor 3 TFF3 (11-12). Assays to detect proteins which are stable compared to other cell products such as RNA were chosen for their applicability to routine clinical diagnostic pathology laboratories.

Next we examined the feasibility and acceptability of using the Cytosponge in primary care (www.beststudy.org.uk), (13-14). 501 patients (mean age, 62 years) with a history of reflux disease were recruited from 2,692 eligible general practice patients giving an uptake of 18.7% (similar to participation rates in previous primary care endoscopic studies) (15-16). Only 3/504 (<1%) were unable to swallow the device (after 3 attempts) and they were from 2 different surgeries. There were no serious adverse events (bleeding, perforation, detachment of the sponge from the string, effects on the airway) and no ill effects reported after the procedure.

Cytosponge results were compared with endoscopy as the gold-standard with a compliance of 92%. In this population the prevalence of BE containing specialized intestinal metaplasia was 3.0%, in keeping with other non-UK population data (16-19). Although not powered to determine accuracy of the test as a primary outcome measure, the Cytosponge test detected BE (TFF3) with a sensitivity and specificity of 73%-90% and 95% respectively. Hence, this pilot study has demonstrated that the Cytosponge is simple and safe enough to be applied to the primary care setting to diagnose Barrett's oesophagus. However, more robust estimates of sensitivity and specificity are required. Furthermore, using the same Cytosponge samples, we have demonstrated that conditions such as *Helicobacter pylori*, oesophagitis, eosinophilic oesophagitis; or squamous atypia, suggesting squamous dysplasia and candidiasis can also be diagnosed and preliminary data suggest that there is high accuracy (90-100%) compared with the current gold standard, although further data is required to substantiate this finding.

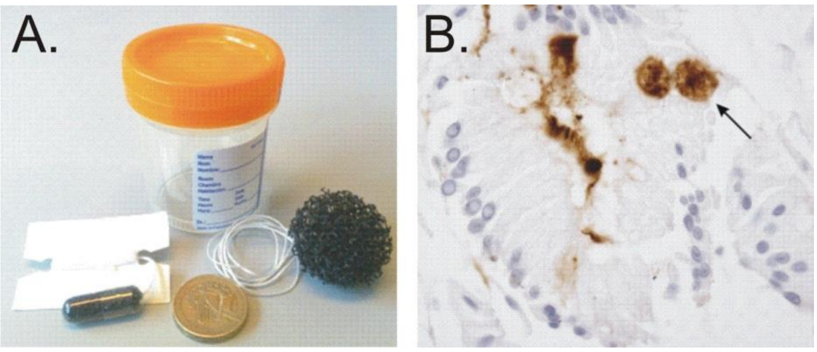

Figure 1: Cytosponge within the capsule and expanded (A) and representative picture of positive TFF3 staining in a sample from a patient with BE (B)

Biomarkers for risk stratification

BE progresses to adenocarcinoma via a sequence of cellular changes which can be detected by the pathologist under the light microscope. These changes are described by pathologists as metaplasia (BE with no abnormal cell changes -dysplasia (alterations in the tissue architecture such that there are more cells dividing and less organisation of the tissue) -adenocarcinoma sequence. The conversion from BE to cancer occurs at a rate of 0.6% per year (20). Once dysplasia is present the likelihood of cancer developing increases. Therefore, in order to avoid placing an undue burden on endoscopic surveillance, the ideal screening test should also stratify patients according to their likelihood of progressing to AC. This needs to be done as accurately as possible.

Dysplasia is a highly subjective diagnosis and pathologists commonly do not agree on the precise grade of dysplasia. Also dysplasia cannot be graded using the kind of superficial cell specimen obtained by the Cytosponge. Therefore, more objective measures are required. There are a number of molecular changes which occur in the progression towards cancer which could be used as stratification tools if applied to the Cytosponge.

These include changes in DNA content of the cell (DNA ploidy), and changes in tumour-suppressor genes called p16 and p53. Recent research suggests that in patients with abnormalities in all three of these molecular parameters the risk increases by 38.7 (95% CI 10.8-138.5; $p<0.001$) compared to those individuals that do not have any of these abnormalities (21-22). In addition, another panel of altered chemical groups (methyl groups which have the effect that genes are not expressed in the cell) in 8 genes are also promising (23-24). However, the measurements required (assays) for these analyses are highly technical. Alternative methods have been developed which do not rely on frozen biopsies and could be applied to preserved cells such as those obtained from the Cytosponge. These include methods for determining DNA content which analyses the size and density of the nucleus (image cytometric analysis), (25-26) and fluorescent probes to count the number of chromosomes (centromeric FISH probes). Alterations in tumour suppressor genes can be determined using antibodies which are routine in NHS pathology laboratories (immunohistochemistry) (27). In addition, we have found that detection of proteins necessary for proliferation such as Cyclin A and MCM2 detect >90% high grade and adenocarcinoma cases (12, 28). Since these tests are all markers which specifically detect cell surface abnormalities using routine techniques they should be ideally suited to the Cytosponge. Furthermore, the methylation panel can be applied to DNA extracted from the cell pellet.

Risks and benefits for participants:

Patients taking part in this study will be carefully evaluated for the presence of BE if they are being seen for the first time. Patients with known BE will be very carefully monitored for the presence of dysplasia. The endoscopists will be using strict protocols and the biopsies will be evaluated by expert trial pathologists for this study as compared to normal practice. The Cytosponge samples will also be evaluated for molecular markers that may suggest an increased risk for cancer progression. The information gained from these analyses will help determine which patients need more careful monitoring or treatment in the future The results of these molecular analyses will not alter their management in this trial.

Since all patients taking part in the trial would be having an endoscopy in any case the risks are extremely low. Taking part will involve an additional test the Cytosponge which data from our previous studies (13-14) suggests is well tolerated with no adverse events and extremely safe.
The Cytosponge test will not provide any immediate benefit to the participants and will incur an additional 30 minutes for the information to be given and for the procedure to be carried out. However, by participating in this study, they will be helping to provide useful information for the assessment and treatment of this disease. In the future this test may prevent unnecessary endoscopy and improve our ability to predict patients at higher risk for developing cancer.

2. Study objectives and purpose

The purpose of the study is to obtain more accurate data on the potential of the Cytosponge as a screening modality (in conjunction with TFF3 for BE), and to find out its potential to determine the risk of cancer progression (in conjunction with biomarkers of risk).

The objectives of the study are:
a) to test the safety and efficacy of the Cytosponge and in particular whether the Cytosponge in conjunction with immunohistochemistry for TFF3 is able to diagnose BE in patients with known BE compared to patients with symptoms of indigestion (dyspepsia) or reflux and,
b) to test whether biomarkers, indicative of the risk of progression to adenocarcinoma, correlate with the dysplasia status of the patients.

3. Study design

Primary Outcome

- Performance and safety characteristics of the Cytosponge test.
- Effectiveness of the Cytosponge for diagnosing BE compared with endoscopy, including specificity (from controls) and sensitivity (from cases).
- For patients with BE, the ability of Cytosponge biomarkers to risk stratify patients in comparison with dysplasia grade obtained from endoscopic biopsies.

Secondary outcomes

- Differential sensitivity of screening BE with dysplasia (low and high grade) compared to non-dysplastic BE.
- Determine the reproducibility of the Cytosponge result by repeated testing in a subset of individuals.
- Logistics of high-throughput sample processing and automated analysis of Cytosponge specimens for use in routine NHS or other health care settings.
- Assess the diagnostic accuracy of the Cytosponge for diagnoses (e.g.*Helicobacter pylori*, oesophagitis, eosinophilic oesophagitis or squamous atypia, suggesting squamous dysplasia and candidiasis) compared to endoscopy.

Potential for bolt-on studies and translational research

- Surplus material will be used for testing emerging biomarkers.
- Opportunity to re-contact participants in this study in relation to other cancer prevention behavioural interventions e.g. smoking cessation, obesity reduction or chemoprevention.

Duration

The total duration of the study will be 3 years, including recruitment of patients in the first 12 months. The time of involvement for an individual will depend on when in the time-line of the study they were recruited. Patients with Barrett's oesophagus may have repeated Cytosponge tests and endoscopy as clinically indicated during the entire duration of the study. The majority of controls will only have one procedure unless they have a false positive Cytosponge test where they will be called for a repeat test. We would like to flag all participants with the office for national statistics for cause of death for 10 years beyond the end of the study.

Sample Size:

The main goal is to obtain accurate estimates of the sensitivity and specificity of the screening tool. For this we plan to obtain between 500 and 700 individuals with Barrett's oesophagus (cases) including at least 100 with low-grade dysplasia and, if possible, 100 with high-grade dysplasia. We plan to recruit roughly equal numbers of controls to cases.

Flowchart of the study

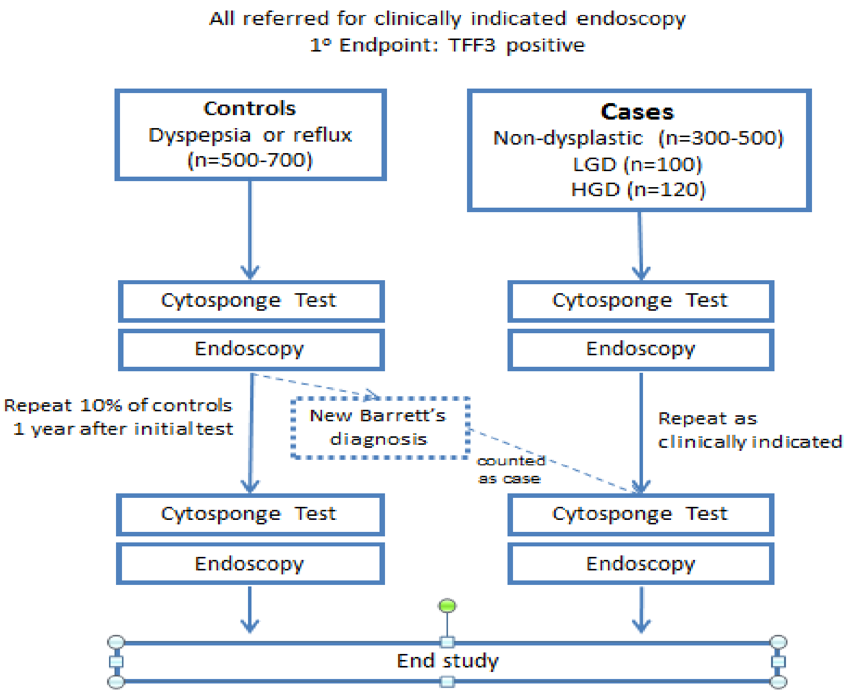

Figure 1: Flowchart of the trial

Each participant will be assigned a unique study number. The individual(s) processing the samples, performing the staining and scoring for TFF3 will identify patients using these study numbers and will be blinded to the identity and the case-control status of the participants.

After the participants' samples have been scored for TFF3, the cases will then be tested for the biomarkers of risk of progression. The individual(s) running the tests will be blinded to the dysplasia status of the patients.

In order to determine the reproducibility of the Cytosponge test findings and to obtain information on how often the Cytosponge should be repeated, control patients with a false positive result will be asked to repeat the Cytosponge test +/- endoscopy a year after the initial test.

For participants with known BE, they will have a repeat endoscopy at intervals determined by the grading of dysplasia on their endoscopic biopsies. This is according to standard clinical practice. For these individuals the Cytosponge test will be repeated each time they attend for an endoscopy to compare the findings from the two procedures.

a. Cytosponge

The device consists of a spherical 3 cm (2.8 and 2.5 cm only available for a pilot of 20 patients at Cambridge) diameter reticulated polyester foam compressed and encapsulated in a standard gelatine capsule (size 00). The sponge is attached to a length cord (Astralen, braided synthetic non-absorbable suture) which passes out through the capsule. In use the capsule is swallowed and allowed to reach the stomach while remaining attached to the cord which is held onto by the patient or clinician. In the stomach the capsule dissolves allowing the sponge to expand to its full size. It is then withdrawn using the cord, and as it does so collects cells from the lining of the oesophagus. Ultimately these cells are retrieved from the sponge and analysed for evidence of pathology. This device previously received a letter of no objection by the MHRA for use in the BEST pilot trial (LRQ 0939857) but it is not CE marked. The device used in this trial will be identical to that used in the BEST pilot except for the substitution of the previous cord with Astralen which is a more economical alternative which has equal tensile strength. The knot will be a Poacher's noose which is designed to reduce the risk of loosening. and is easier to tie compared to the knot in the original application therefore reducing the risk of detachment. The Cytosponge device will be supplied to the participating centre and they will be responsible for storage and accountability of the device. The Cytosponge product lifetime/Use by date will be confirmed on the product packaging.

b. Study procedures

Recruitment of Participants and Informed Consent Procedure

All participants will be identified from the list of patients invited to attend for clinically indicated endoscopy either as part of their BE surveillance or to investigate symptoms of reflux or dyspepsia. The research nurses at each centre (Cambridge, UCL, Nottingham and Newcastle, and all new sites) will check the endoscopy referrals at least twice a week to maximise the identification of eligible participants.

Individuals meeting the inclusion criteria (see below) will be contacted prior to their endoscopy as to ascertain their willingness to participate. For most participants this will be a letter sent to them at least 48 hours prior to their endoscopy appointment which will include a participant information sheet (PIS) and a consent form. For individuals being seen in the clinic researchers may also discuss the study in person and provide them with the study pack.

For these participants willing to take part in the study an appointment will be arranged for them to have a Cytosponge test just prior to their endoscopy procedure. A standard appointment letter along with the study consent form will be sent out to the participant. At the appointment participants will be offered the opportunity to ask questions.

Participants will be consented on the day of their endoscopy appointment by a research nurse who has been trained in the informed consent procedure and may have the opportunity to talk to the PI or a nominated health professional, should they have any further questions.

The study participants (controls and cases) will consent to participate in the study for 3 years i.e. this will allow for repeat CytoSponge test(s) or not. The controls will all undergo a single Cytosponge test at entry but only controls with a false positive test will be invited for a follow-up Cytosponge test a year after entry into the study. All cases will have repeat Cytosponge test(s) when attending for a follow-up surveillance endoscopy according to standard clinical care, within the duration of the study. This will enable us to monitor alterations in biomarker status according to the grade of dysplasia. In addition, all consented participants will be flagged for 10 years once the study ends.

It is routine practice for patients having an endoscopy to consent to the procedure. This consenting procedure will carry on as normal and in parallel to consent for the study.

Baseline information

The participant will provide information on demographics, clinical exposures (alcohol, tobacco, drugs), have measurements of weight, height and waist:hip ratio taken and they will also complete a validated reflux questionnaire (29). The data will be entered by the study nurse at the local hospital using a custom-made application with secured access via the web, the BEST 2 app.

Blood samples

A blood sample will be taken at baseline as a reference for DNA analyses on the tissue and for future biomarker studies. A total of 10 mls (e.g. 2 teaspoons) of blood is taken, 5 mls in EDTA and 5 mls in sodium citrate (Coagulation). The EDTA (Ethylenediaminetetraacetic acid) sample is frozen (-80 freezer) within 6 hours of the sample taken. The sodium citrate sample is centrifuged at 3000 rpm for 10 minutes and then the plasma is pipetted into a new sodium citrate tube and both samples are frozen (- 80 freezer). Standard operating procedures (SOPs)/guidance manual are written to inform the sites on sampling, labelling, storage, collection, transport and delivery of samples.
All samples will be tracked using the BEST 2 app.

The blood sample may also be counted for the Barrett's Oesophagus Gene study and BEST 2 where the participant has agreed to participate in both studies. Both studies are led by the same Chief investigator Prof Rebecca Fitzgerald. The primary aim however is to ensure that the blood sample is taken when the participant attends their BEST 2 appointment for the study.

Cytosponge procedure

The Cytosponge will be administered by the study nurse prior to the participant having the endoscopy and usually on the same day as informed consent i.e. as part of the same visit to hospital.

The capsule along with three quarters of the string (which is bunched together holding it close to the capsule to make it easy for swallowing) is swallowed by drinking a small glass of lukewarm water continuously. The participant is asked to hold the end of the string with the Cytosponge in situ for 5 minutes. This is to allow the outer gelatine cover of the capsule to dissolve in the stomach acid. The sponge contained within expands and is then drawn back by the research nurse up the oesophagus by the attached string, collecting cells as it move upwards.

Once the Cytosponge is withdrawn it is placed in the preservative fluid and the excess string is cut with a pair of scissors and disposed of in the clinical waste. The Cytosponge sample can be kept at room temperature but must be stored in the fridge at $4^{o}C$ within 6 hours. The samples will be sent to the laboratory at Cambridge (MRC/Hutchison Research Centre) to be analysed in batches.
SOPs for labelling, storage, collection, transport and delivery of samples will need to be applied to all samples taken for this study.

A pilot of 20 patients will be recruited to swallow the smaller size Cytosponges (diameter 2.5 and 2.8 cm) The size specification is a range +/- 0.5cm which make a significant difference to the volume. We wished to test the lower end of this range to see if there was any noticeable difference in acceptability. This is not to perform a robust statistical analysis but to help make a final decision on size for the CE marking application in due course. These smaller devices will be 20/1000-1400 devices for the final trial. The cellularity of the sample is noted for all cases and if this is inadequate then such samples are excluded from final analysis. The same criteria will be applied to these smaller devices but it is not anticipated that the cell yield will be affected.
The smaller devices will be randomly allocated to a single size and this noted on the participant study record.

The participants at Cambridge only, will be asked for a throat swab to be taken. The sample should be taken from oropharyngeal region at the back of the mouth and snap frozen immediately afterwards. Cytosponge samples collected in the BEST2 trial offer a novel opportunity to study the microbiome along the entire length of the oesophagus surface epithelium. Since the Cytosponge is withdrawn on a string through the mouth and throat, it will collect microbes all along the length of the tract from mouth to stomach. Therefore, to better analyse and interpret the oesophageal microbiome composition and its function in Barrett's oesophagus, we would like to obtain additional mucosal swab samples from the oropharynx of participants.

Endoscopy procedure

After the Cytosponge test the participants will then undergo their clinically indicated endoscopy. An NHS consent form will be signed prior to this procedure as part of standard clinical practice. The study consent form will have been signed prior to the Cytosponge procedure. The endoscopy will proceed immediately after this NHS consent has been obtained.

During the procedure the endoscopist will note the endoscopic diagnostic landmarks for BE using a standard protocol. The findings will be entered in the endoscopy CRF as part of the BEST 2 app. As well as any clinically indicated biopsies some additional samples will be taken for research purposes providing the patient tolerates the procedure: one brushing (a small brush is rubbed against the oesophageal lining) from the squamous oesophagus will be taken from all participants and another from an area of Barrett's mucosa will be taken from those with BE. The brushing will be collected for biomarker evaluation to compare with the Cytosponge specimen as previously described (12, 28). In participants with known BE, biopsies in all 4 quadrants will be taken every 2 cm according to surveillance guidelines. In addition, one snap frozen biopsy per level of Barrett's oesophagus will be collected. In patients with a Barrett's Oesophagus segment greater than 8 cm the number of Barrett's samples taken may be abbreviated according to how well the patient tolerates the procedure. Participants (including controls) will have biopsies collected from 2cm above the squamo-columnar junction (SCJ). These samples are summarised in the table below.

	**Cases**	**Controls**
Blood sample	10ml	10ml
Cytosponge	In Surepath preservative	In Surepath preservative
Endoscopic brushings	In Snap Frozen<br>- BE: 2cm above GOJ *<br>- NE: 2cm above BE	- *In Snap Frozen From 2cm above GOJ*
Endoscopic biopsies	Snap frozen<br>- Duodenum *<br>- 1 per BE level (may be abbreviated if segment >8cm and procedure poorly tolerated<br>- NE 2cm above BE *	Snap frozen<br>- Duodenum *<br>- NE 2cm above GOJ*
	In formalin<br>- Gastric cardia (GC) (from 2cm below the GOJ)	In formalin<br>- Gastric Cardia (from 2cm below the GOJ)

Table 1: research samples to collect for cases and controls (GOJ: gastro-oesophageal junction; SCJ: squamo-columnar junction; NE: normal squamous oesophagus).

(*) These samples may be optional if the patient is not tolerating the procedure.

Please note: Where sites are unable to provide liquid nitrogen or dry ice for snap freezing sample collection requiring snap freezing will be omitted.

Brushes may be omitted if the unit is unable to provide the equipment for collection of these samples.

SOPs/Procedure manual for biopsy sampling, labelling, storage, collection, transport and delivery of samples will need to be applied to all biopsies taken for this study.

Follow-up visits
It is the responsibility of the local PI to schedule the next follow-up visit within the BEST 2 app.

	**Cases**	**Controls**
Blood sample	-	-
Cytosponge	In Surepath preservative	In Surepath preservative
Endoscopic brushings	-	-
Endoscopic biopsies	Snap frozen<br>1 per BE level (may be abbreviated if segment >8cm and procedure poorly tolerated	-

Table 2: research samples collected for cases and controls at follow up visit

Cases
As mentioned briefly above, participants with BE will have repeat Cytosponge testing at each endoscopic surveillance procedure during the course of the study. It is of interest to know whether the failure of the Cytosponge to detect BE is a "random event" or whether it is related to the particular participant and their BE. For this reason the sensitivity of the Cytosponge in cases who had a previous negative "screen" will be evaluated. It is also of interest to learn whether the Cytosponge could be used for surveillance (instead of endoscopy) to monitor possible progression in patients with BE. For this reason cases will be asked to undergo Cytosponge collection with biomarker testing at each (routine) endoscopy appointment during the study. The interval will depend on their dysplasia grade at the time of their previous endoscopy in accordance with surveillance guidelines. Each BE participant will be invited for repeat endoscopy according to local standard in patient management.

Controls
In addition all control participants with a false positive test will be invited to attend for a repeat Cytosponge after an interval of 1 year. The acceptance rate for a repeat test will be useful information on the acceptability of screening. We also wish to learn about the specificity of the Cytosponge in controls who have been previously screened.

Initially we aim to obtain repeat samples at approximately 12 months from Controls who tested false positive. Since it is desirable to use endoscopy as a gold-standard at this stage in the evaluation

of the Cytosponge, but preferable not to do endoscopy unless clinically indicated, we shall aim to recruit these controls from among participants who are booked for a clinically indicated endoscopy approximately 12 months after entry in the study. If there are too few such controls, others will be invited to have a second Cytosponge test. Such participants will also be offered endoscopy, but if a participant has a second Cytosponge test but declines endoscopy it will not be considered a protocol violation.

In patients (Cases and Controls) where the cytosponge has been analysed and found to have an excessive number of eosinophils this may be indicative of a diagnosis of eosinophilc oesophagitis. Similarly if squamous atypia is diagnosed on the Cytosponge this may be indicative of squamous dysplasia. Both of these are clinically relevant entities. In such cases the coordinating site (Cambridge) will contact the managing clinician. If this respective diagnosis is not already known then a repeat endoscopy will be performed at the discretion of the managing clinician if this is felt to be relevant to patient management. At repeat endoscopy, biopsies will be taken according to standard clinical practice. A repeat Cytosponge test will be optional.
Patients will be asked to contact their PI or research nurse if they have any adverse events in the 2 weeks following the procedure. They will also be asked whether they are willing to be contacted for any of our future research, not necessarily related to this study.

Patients initially recruited as controls and then found to have BE, confirmed from pathology results at baseline will be transferred to the cases arm of the trial. New patients will be recruited to make up the numbers for the Control arm of the study.

Flagging

Patients will be asked to consent to being "flagged" so that we can monitor whether they subsequently develop BE with/without dysplasia. We would like to use local records from the endoscopy clinic (and pathology) as well as national records of cancer registration i.e. NHS Information Centre (NHS IC).

The NHS IC recommends collecting the following details to enable best auto-match rates: NHS number, full surname, full forename, date of birth and full postcode is also useful. Full address details, email address and telephone and mobile numbers will also be collected so that consented patients can be re-contacted in relation to other cancer prevention behavioural interventions e.g. smoking cessation, obesity reduction or chemoprevention.

The above information will be collected from the consenting patients via the BEST 2 app and this information will be stored separately from their clinical information.

Flagging activities are anticipated to start at the end of the study and will last for 10 years.

Cytosponge processing

All Cytosponge and research endoscopic biopsies will be couriered to the Fitzgerald laboratory, at the MRC Cancer Cell Unit on a regular basis. Cytosponge specimens will be processed in conjunction

with the Cambridge University Hospitals' NHS Foundation Trust tissue bank which is accredited to GLP standards (11). Samples will be processed to a paraffin embedded cell clot, sectioned and evaluated for the Barrett's diagnostic biomarkers (TFF3) using a DAKO autostainer according to GCP standards. Samples will be scored in a binary fashion (positive or negative) as already optimised from the pilot study. A screener will identify areas of interest for the expert GI cytologist (Maria O'Donovan) to verify. The Cytosponge samples will be processed within a 4 /6 week period so that positive samples can then be further processed for additional biomarkers as described below. Although timely processing will ensure that the study completes on time the results of Cytosponge samples will not affect patient clinical care and therefore there is some flexibility in the timeline.

Samples (from any patient whether known BE participants or controls) with a positive result for the Barrett's diagnostic biomarkers will have additional sections cut from the existing endoscopic biopsies and Cytosponge sample for risk stratification biomarkers. Mcm2, cyclin A and TP53 can be performed on the BOND autostainer. The ploidy analysis (again only if positive for BE markers) will be performed using FISH (Krishnadath laboratory, Amsterdam) and methylation will be performed using methylation specific PCR (23), (Fitzgerald laboratory). The biomarkers will be scored according to previously optimised protocols (11-12, 25, 28). These data will be compared to the degree of dysplasia determined from endoscopic biopsies.

The laboratory trial manager will determine whether to proceed with additional biomarkers based on the TFF3 status.

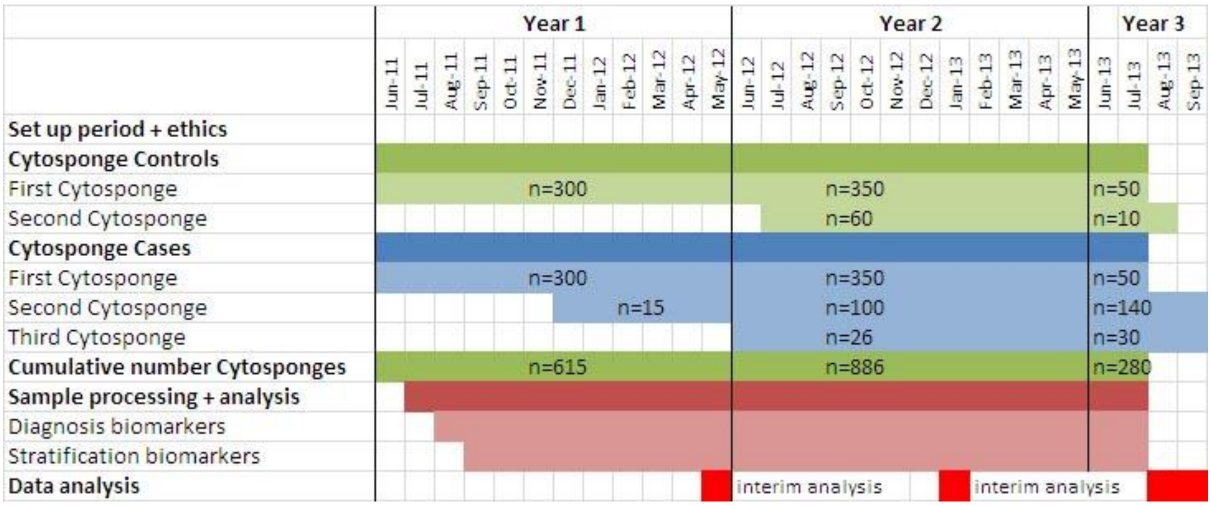

Figure 2: Timeline of the study (n indicated the number of patients to recruit for each arm). Only cases and 10% of controls will have repeat sponges.

4. Data Collection

Data will be collected electronically from the participating sites using BEST 2 app. PDF copies of the CRFs will also be made available to the PIs which can be printed. Data should be recorded directly and legibly onto the BEST 2 app.
In exceptional circumstances completed paper CRFs may be returned to the Study Coordinator.

Where papers CRFs are used, corrections should be made legibly by approved personnel and initialled, signed and dated. Original entries should be crossed through with a single line so that they are still legible. Correction fluid or covering labels must not be used. Original paper CRFs will be kept at the local site whereas copies will be sent to the Study Coordinator.

Below is a summary of the Case Report Forms (CRFs) or questionnaires that will be collected as part of this study.

Co-morbidities

Current co-morbidities details will be obtained from the patient's medical records or directly from the patient on the day of their endoscopy appointment. Co-morbidities are listed on the CRF in a drop down menu and range alphabetically from anaemia to vascular heart disease. Each co-morbidity will need to be added individually from the list. If the list does not contain an option then select other. A user manual will be provided for BEST2 app at each centre.
It is the responsibility of the local PI or designated healthcare professional at each site to update the medication history Case Report Form (CRF) at baseline and follow-up visits.

CONTROLS				
	**Baseline**	**Year 1**		
	**1st swallow**	**2nd swallow**		
**Scheduled CRFs**				
Eligibility (incl/excl criteria)	X			
Baseline CRF including Demographics, General Information, Smoking, Alcohol, Family History, Symptoms, Comorbidities, Other Study(ies) Participation	X			
Medical Events	X			
Medication History	X	X		
Blood sample (5mls EDTA and 5mls sodium citrate)	X			
Cytosponge procedure (1)	X	X		
Endoscopy (mucosa AND oesophagus) procedure (2)	X	X (Optional)		
Biomarker work-up (diagnostic/prognostic) (1)	X	X		
Pathology (clincial+consensus) (2)	X	X		
Follow-up CRF includinggeneral Information, Family History, Symptoms		X		
Unscheduled CRF				
Schedule next visit				
AE /SAE (3)				
Early withdrawal				

CASES			
	Baseline	As indicated (Y1-2)	As indicated (2)- Optional
	1st swallow	2nd swallow	3rd Swallow
**Scheduled CRFs**			
Eligibility (incl/excl criteria)	X		
Baseline CRF including Demographics, General Information, Smoking, Alcohol, Family History, Symptoms, Comorbidities, Other Study(ies) Participation	X	X	X
Medical Events	X	X	X
Medication History	X	X	X
Cytosponge procedure (1), including blood sample (5mls EDTA and 5mls sodium citrate)	X	X	X
Endoscopy (mucosa AND oesophagus) procedure (2)	X	X	X
Biomarker work-up (diagnostic/prognostic)	X	X	X
Pathology (clinical+consensus) (3)	X	X	X
Follow-up CRF including General Information, Family History, Symptoms		X	X
**Unscheduled CRFs**			
Schedule next visit			
SAE (3)			
Early withdrawal			

1) x1 sample, analysis in Cambridge only
2) controls: minimum x2 samples and 5x sets of results: local + 3 sets from separate study pathologists + consensus opinion
3) cases: minimum x5 samples and 5x sets of results: local + 3 sets from separate study pathologists + consensus opinion

Table 3: Overview of data collection

5. Selection and withdrawal of participants

a. Recruitment

500–700 individuals with known BE (cases) undergoing surveillance, including those referred for further evaluation and management of high grade dysplasia (HGD) and low grade dysplasia (LGD) will be recruited. There will be a purposeful sampling of patients to ensure an adequate number of dysplastic patients (10% cohort with HGD and 20% with LGD) are recruited in order to evaluate the biomarkers.

500-700 individuals referred by their GP for an endoscopic evaluation of reflux or dyspepsia (controls) will also be invited to attend.

Patients whose Cytosponge failed to fully expand will not contribute to the analysis of sensitivity and specificity or the biomarker discovery. However such patients should be included in the study under the classification of 'sponge failures' and asked to swallow the Cytosponge device again at their next endoscopy appointment providing it is within the time scale of the study.
Controls later found to have BE will be transferred to the Cases arm of the trial and replaced. Extra patients will be recruited to make up the numbers.

Inclusion

- Any participant 18 years and above clinically fit for an endoscopy with Barrett's oesophagus (Cases) with or without upper GI symptoms
- Any participant 18 years and above clinically fit for an endoscopy with upper GI symptoms of reflux or dyspepsia as an indication for endoscopy/gastroscopy (Controls)
- Ability to provide informed consent
- Patients who have undergone Endoscopic mucosal resection (EMR) for high grade dysplasia and due for repeat endoscopy with residual Barrett's oesophagus
- Patients who have undergone Radiofrequency ablation (RFA) ≥ 6months for high grade dysplasia and due for repeat endoscopy with residual Barrett's oesophagus providing there is no stricture.

Exclusion criteria

- Individuals with a diagnosis of an oro-pharynx, oesophageal or gastro-oesophageal tumour, or symptoms of dysphagia,
- oesophageal varices, stricture or requiring dilatation of the oesophagus
- on anticoagulation therapy/medication (warfarin, clopridogrel,heparin or tinzaparin) on the day of their procedure
- *Individuals who have had a myocardial infarction or any cardiac event less than six months ago.*
- *Individuals who have had a cerebrovascular event < 6 months ago where their swallowing has been affected*
- *Patients who have had previous treatment such as Photodynamic therapy (PDT) or Radio Frequency Ablation (RFA)in the last 6 month*
- Participants who are unable to provide informed consent.
- Participants under age 18.
- Participants who exclude beef from their diet as the gelatine is beef based. This can be discussed with the patient.
- Endoscopy is generally avoided in pregnant women and therefore it is unlikely that any pregnant women will be included although pregnancy would not be an absolute contraindication. Pregnancy/ pregnancy test will not be recorded as part of the trial.

b. Withdrawal

Any participants who had a severe reaction to the Cytosponge will not be invited to participate in the second round.

Each site lead (PI) is qualified to make medical decisions concerning withdrawals from the study.

6. Assessment of efficacy

Cytosponge preparation and TFF3 staining will be done in the Tissue Bank within the department of Histopathology of Cambridge University Hospitals NHS Foundation Trust according to GCP and GLP guidelines. Samples will be prepared within 2-4 weeks of the sample collection. Cytosponge samples can be transported at room temperature and be placed in the fridge at 4°C until processed.
A single slide will be cut from each block for TFF3 staining. 10 additional slides will be cut simultaneously for biomarker assays.

TFF3 staining will be scored in a binary fashion (positive or negative) by an individual trained by an expert gastrointestinal histocytologist (Maria O'Donovan). 10% of negative slides and all positive slides will be review by the expert gastrointestinal histocytologist (Maria O'Donovan).

The biomarker data will be scored but the precise algorithms will be determined during the pilot phase of the first 50 patients recruited.

7. Assessment of safety

This will be a major objective as the Cytosponge is a novel device. SAEs and AEs CRFs will be reported following the sponsors (Cambridge University Hospitals NHS Foundation Trust) procedures.

Definitions

An ***Adverse Event (AE)*** is any untoward occurrence in a study participant which does not necessarily have a causal relationship with the study treatment or investigations. An AE can be any unfavourable and unintended sign, symptom or disease temporally associated with the study, whether or not related to the study.

A ***Serious Adverse Event (SAE)*** is an untoward medical occurrence that:

1. Results in death
2. Is life-threatening
3. Requires hospitalisation or prolongation of existing hospitalisation
4. Results in persistent or significant disability or incapacity
5. Consists of a congenital anomaly or birth defect
6. Is otherwise considered medically significant by the investigator (eg. a further procedure is required for the patient).

Recording of serious adverse events (SAEs)

All participants will have Alert stickers on their medical records to indicate that they are participating in the study. The trial Information will be recorded in their notes along with contact details should an AE or

SAE event occur. Each patient will be provided with after care forms and contact details indicating clearly who to contact in the unlikely event of an AE.

AEs (including SAE) occurring within 2 weeks of the procedure only will be recorded and investigated (since previous studies using this device and endoscopy studies have found to occur within 2 weeks after the procedure).

The PI at that site will assess each AE/SAE for relatedness (resulted from the procedure) and unexpectedness (see below for a list of expected events). A copy of the report will be placed in the patient medical records and entered on the BEST2 app.

In the case of a SAE, the SAE CRF available on the BEST2 app. will be completed by the PI within 24 hours of the discovery of the event. The SAE CRF will also be printed and a signed copy sent (faxed 01223 348071 or scanned) immediately to the coordinating centre where it will be checked for completeness by the study coordinator, and queried with PIs if necessary.

The study coordinator will forward all SAEs to the CI and sponsor as soon as possible. The CI will review each SAE, and if it is both related and unexpected, the CI should report it to the main REC and the MHRA within 15 days of becoming aware of the event, using the NRES report of serious adverse event form. Procedure-related deaths are not expected, but if any do occur this will lead to review of the study continuation. An urgent meeting with the management committees will be arranged immediately to discuss the safety of using the Cytosponge.

Events relating to the Cytosponge or endoscopy could technically include:

- Cytosponge detached from the string while in the patient's oesophagus/stomach (SAE)
- inability or difficulty to remove the Cytosponge (SAE)
- laceration at the back of the throat (AE or SAE depending on Bleeding and size of the laceration)
- perforation or tear of the oesophagus (SAE)
- bleeding form biopsy site (SAE)
- obstruction on breathing or airway as a result of the Cytosponge (SAE)

If the Cytosponge detaches from the string or if the Cytosponge cannot be removed, the recommended approach is to remove the Cytosponge by endoscopy within 3 hours of the ingestion. Since all patients will undergo an upper GI endoscopy following the procedure this will not be a problem. More than 500 individuals have swallowed this device in a previous MHRA approved trial and none of these events occurred therefore the risk associated with the study is minimal.

8. Statistics

a. Planned statistical analysis

All individuals offered the Cytosponge will be included in analyses of acceptance and adequacy of sampling. All individuals with adequate Cytosponge samples and endoscopy (within 48 hours of Cytosponge) will be included to determine the sensitivity and specificity for diagnosis of Barrett's from the TFF3 data. 95% confidence intervals will be used.
Recursive partitioning and ROC curves will be used to select cut of values for biomarkers of interest. Multivariable analysis will be performed using logistic regression. Inference in the biomarker discovery phase of the study will be assessed using cross-validation. For each case, a p value < 0.01 will be required for significance to allow for multiple testing.

Interim analysis of the data is planned as indicated in figure 2.

b. Sample Size Consideration:

The primary objectives of the statistical analysis are:

(1) To estimate the overall sensitivity of Cytosponge to Barrett's oesophagus with accuracy (95% confidence interval) of between +/-1.5% and +/-3.0%. With a sample size of 500 to 700 individuals with Barrett's (cases) we could achieve. Table 1 presents the approximate width of 95% confidence intervals for different underlying sensitivities.

Sensitivity	Sample size 500	Sample size 700
95%	+/- 1.9%	+/- 1.6%
90%	+/-2.6%	+/-2.2%
85%	+/-3.1%	+/-2.6%

Table 4: Width of 95% CIs depending on underlying sensitivity and sample size

(2) To estimate the overall specificity in individuals without Barrett's oesophagus (controls) with 95% confidence intervals of between +/-1.0% and +/-2.0%. Greater accuracy in specificity estimation is desirable as a decrease of 1% in specificity means that an extra 1 in 100 people screened would have a positive screen and (potentially) an unnecessary endoscopy and biopsy.

Specificity	Sample size 500	Sample size 700
98%	+/- 1.2%	+/- 1.0%
97%	+/-1.5%	+/-1.3%

95%	+/- 1.9%	+/- 1.6%
93%	+/-2.2%	+/-1.9%

Table 5: Width of 95% CIs depending on underlying specificity and sample size

(3) To compare sensitivity to high grade dysplasia relative to low-grade dysplasia and non-dysplasia, and to identify a biomarker for high-grade. We aim to include 120 for cases with high grade dysplasia and would be willing to collect up to 700 cases in total. In general we would expect about 10% of cases to have high-grade dysplasia, but this should be an enriched population. There are likely to be up to twice as many cases with low-grade as high-grade dysplasia. As a result, we expect the number of cases without dysplasia to be around 350-500.

Proportion	Sample size 75	Sample size 100	Sample size 350	Sample size 500
98%	+/-1.6%	+/-1.4%	+/-0.7%	+/-0/6%
95%	+/-2.5%	+/-2.2%	+/-1.2%	+/-1.0%
85%	+/-4.1%	+/-3.6%	+/-1.9%	+/-1.6%
80%	+/-4.6%	+/-4.0%	+/-2.1%	+/-1.8%

Table 6: Width of 95% CIs depending on underlying proportion and sample size: Sample sizes of 75 and 120 for high-grade and low-grade dysplasia, sizes of 350 and 500 for no dysplasia; For a biomarker the proportions could be sensitivity for high-grade dysplasia and specificity for Barrett's without dysplasia.

If the sensitivity of the screen is 90% in Barrett's without dysplasia (n=350) and 99% in Barrett's with high-grade dysplasia (n=100), one would have 89% power for a two-sided Fisher exact test with alpha of 0.05. For a biomarker aimed at distinguishing high-grade dysplasia from Barrett's without dysplasia, we may wish to test the hypothesis that the odds ratio is greater than some value (such as 10) rather than simply testing whether or not it is equal to 1.0. If the true sensitivity is 85% and the true specificity (in those without dysplasia) is 20% (corresponding to an odds ratio of 22.67), then the power to show that the 95% confidence interval for the odds ratio excludes 100 is 81% (obtained by simulation).

(4) To estimate the specificity on repeat testing for controls with false positive results (TFF3 positive). All false positive controls will be invited for rescreening in order to determine whether the specificity on repeat testing is close to that in all controls initially or whether the majority remain false positives.

(5) To estimate the sensitivity on repeat testing for cases with false negative results. All false negative cases will be invited for rescreening in order to determine whether the sensitivity on repeat testing is close to that in all cases initially or whether the majority remain false negatives.

(6) To estimate the sensitivity and specificity of correlation of risk progression biomarkers with routine diagnosis of dysplasia on biopsies by performing repeat Cytosponge testing in cases when they attend for follow-up surveillance endoscopy

(7) To monitor the safety of testing an urgent teleconference of the management committee will be organised upon notification of a second SUSAR (within 24 hours of swallowing the Cytosponge) among the first 300 participants or a third within the first 600 or a fourth at any point in the study. This is NOT a formal stopping rule, but is a pragmatic rule for discussing the safety of using the Cytosponge in general practice (in this study it will only be used in an endoscopy unit).

(8) Stratification: reassessment of sample size

Another aim of the BEST2 study is to explore the diagnostic ability of Cytosponge for dysplasia. In our interim analysis based on 74 NDBE (Non dysplastic Barrett's Oesophagus), 17 LGDs (Low Grade Dysplasia), 5 at least LGDs and 24 HGDs (High Grade Dysplasia), we found that the specificity for a diagnosis of BE was 74%, the sensitivity to any dysplasia was 85% and the sensitivity to HGD was 92%. We feel that an accuracy of +/-5.0% would be sufficient for this study. We could achieve this goal by recruiting 300 NDBEs, 100 LGDs and 120 HGDs. Based on the proportions of LGD, HGD, and NDBE in our current cohort, the expected sample size for NDBE, LGD and HGD could be achieved by continuing to recruit up to 550 BE but with fewer controls

9. Termination of the study

The study participants (controls and cases) will consent to participate in this study for 3 years as well as being flagged for another 10 years i.e. this will allow for repeat Cytosponge test(s) or not. All controls will undergo a single Cytosponge test at entry but only those with a false positive test will be invited for a follow-up Cytosponge test 1 year later. The cases will have a repeat Cytosponge test(s) following standard care practice.

The study will end when data from all biomarkers has been collected. This will be after the last participant attends their last visit for a Cytosponge test.

No specific medical care should be required by participants at the end of the study. Data will not be used to modify patient's management which will be determined from the histopathological assessment of biopsies according to standard clinical practice. However, particularly worrisome biomarker data or cytological features will be reviewed carefully by M O'Donovan (Consultant Histopathologist) and RC Fitzgerald (CI) and patients may be recalled under the NHS for repeat endoscopy and biopsy is there is any suspicion of dysplasia or cancer

10. Quality control and Quality assurance for sample processing

All biopsy processing and immunostaining for TFF3 will be carried to GCP and GLP standards in a dedicated histopathology unit in Addenbrooke's hospital. The other biomarkers will be performed according to standards from the published literature. These are not currently used as diagnostic

tests and will be performed in a research laboratory. None of the Cytosponge results will influence patient management.
The Barrett's diagnosis and grade of dysplasia will be reviewed by a minimum of two expert gastrointestinal histopathologists. Maria O'Donovan will train the research assistant scoring the TFF3 staining and will review 20% of samples to ensure adequacy of staining. Consultant Histopathologist, Senior Laboratory Scientist and the research assistant will work in concert to design the scoring system for the markers of dysplasia and 20% of cases will be double scored.

11. Regulatory Authorities

Since the Cytosponge is not CE marked and has been modified with an alternative, reticulated polyester foam and a more economical cord this trial will require MHRA approval and safety will be a major objective of the trial.

12. Ethics

The study will be conducted in accordance with ethical principles that have their origin in the Declaration of Helsinki and are consistent with the Research Governance Framework, sponsor and local R&D policies

The CI will send Annual Progress Reports to the Main REC, and report any serious breaches of the study protocol/research governance within 7 days. Any substantial amendments to the research will be submitted to the Main REC before implementation and copied to the MHRA and R&D, except in the case of urgent safety measures which may be taken without prior approval.

13. Monitoring

The study has been risk assessed to determine hazards to the participants, the trial and to the organisation. The recommendation following this assessment was that of low risk, hence in view of this it is felt that central monitoring will be sufficient and that on site monitoring visits will not be part of routine procedures. However, site visits may be triggered by recurrent AEs in a particular centre, an SAE, recruitment not meeting targets, or for site specific issues.

14. Access to source data/documents

Investigators should make source data (i.e. patients' medical records) and study documents held at their site available for possible monitoring and auditing by the study manager or sponsor. The advisory group will meet on an annual basis to review the conduct and progress of the study.

15. Data handling and record keeping

All patients clinical data will be pseudo-anonymised and stored on the secure database of the BEST2 app. Individual centres will have access to their own data via the password protected BEST2 app. The laboratory data will be entered onto separate pages of the same database.

16. Financing and insurance

The study is CRUK funded and the funds are being administered via the University of Cambridge. All posts on the grant will be University employees and the research will be undertaken at Cambridge University Hospitals NHS Foundation Trust. The University of Cambridge will jointly sponsor this study with Cambridge University Hospitals NHS Foundation Trust.

Dr Rebecca Fitzgerald holds a substantive employment contract with the MRC and devised this study whilst under MRC employ; the MRC will provide indemnity for the design of the protocol.

17. Remit of Trial Management and Advisory Groups

Trial Management Group

The Group will meet in person or by teleconference on at least a 6 monthly basis to review study conduct and progress, and review publications and presentations. This group will comment in detail on study procedures and progress.

18. Trial Advisory Group

The Group will meet periodically to review overall study conduct and progress and to advise on future directions.

19. Publication policy

All study investigators will be named on any peer reviewed publications.

20. References

1. Brown LM, Devesa SS, Chow WH. Incidence of adenocarcinoma of the esophagus among white Americans by sex, stage, and age. J Natl Cancer Inst. 2008 Aug 20;100(16):1184-7.
2. Medical Research Council Oesophageal Cancer Working Group, Surgical resection with or without preoperative chemotherapy in oesophageal cancer: a randomised controlled trial. Lancet. 2002 May 18;359(9319):1727-33.
3. Donaldson L. Chapter 6: A pathological concern: Understanding the rise in oesophageal cancer. Annual Report of the Chief Medical Officer. 2007 2007:http://www.dh.gov.uk/en/Publicationsandstatistics/Publications/AnnualReports/DH_086176.
4. Shaheen NJ, Sharma P, Overholt BF, Wolfsen HC, Sampliner RE, Wang KK, et al. Radiofrequency ablation in Barrett's esophagus with dysplasia. N Engl J Med. 2009 May 28;360(22):2277-88.
5. Pouw RE, Wirths K, Eisendrath P, Sondermeijer CM, Kate FJ, Fockens P, et al. Efficacy of Radiofrequency Ablation Combined With Endoscopic Resection for Barrett's Esophagus With Early Neoplasia. Clin Gastroenterol Hepatol. 2009 Aug 11.
6. UK National Screening Commitee, Criteria for appraising the viability, effectiveness and appropriateness of a screening programme. http://wwwscreeningnhsuk/criteria. 2009.
7. Saeian K, Staff DM, Vasilopoulos S, Townsend WF, Almagro UA, Komorowski RA, et al. Unsedated transnasal endoscopy accurately detects Barrett's metaplasia and dysplasia. Gastrointest Endosc. 2002 Oct;56(4):472-8.
8. Dumortier J, Napoleon B, Hedelius F, Pellissier PE, Leprince E, Pujol B, et al. Unsedated transnasal EGD in daily practice: results with 1100 consecutive patients. Gastrointest Endosc. 2003 Feb;57(2):198-204.
9. Ramirez FC, Akins R, Shaukat M. Screening of Barrett's esophagus with string-capsule endoscopy: a prospective blinded study of 100 consecutive patients using histology as the criterion standard. Gastrointestinal Endoscopy. 2008;68(1):25-31.
10. Fennerty MB, DiTomasso J, Morales TG, Peterson D, Karmakar A, Fernandez T, et al. Screening for Barrett's esophagus by balloon cytology. Am J Gastroenterol. 1995 Aug;90(8):1230-2.
11. Lao-Sirieix P, Boussioutas A, Kadri SR, O'Donovan M, Debiram I, Das M, et al. Non-endoscopic screening biomarkers for Barrett's oesophagus: from microarray analysis to the clinic. Gut. 2009 Nov;58(11):1451-9.
12. Sirieix P, O'Donovan M, Brown J, Save V, Coleman N, RC F. Surface expression of mini-chromosome maintenance proteins provides a novel method for detecting patients at risk for developing adeocarcinoma in Barrett's oesophagus. Clin Cancer Res. 2003.
13. Kadri SR, Debiram I, Lao-Sirieix P, O'Donovan M, Blazeby JM, Males A, et al. A pilot feasibility study of screening for Barrett's esophagus with a novel non-endoscopic capsule sponge device in a primary care setting. Gastroenterology. 2009;136 (Suppl 1):T1877.
14. Kadri SR, Lao-Sirieix P, O'Donovan M, Debiram I, Das M, Morris H, et al. Non-endoscopic screening test for Barrett's oesophagus: acceptability and accuracy in a primary care setting BMJ 2011;342:d543:doi:10.1130/bmj.d543
15. Wong A, Lovat L, Burnham RW, Hackshaw A, Fitzgerald RC. Large-scale prospective study reveals novel risk factors for Barrett's oesophagus. Gut. 2007;56(supp II):A8.
16. Ronkainen J, Aro P, Storskrubb T, Johansson SE, Lind T, Bolling-Sternevald E, et al. Prevalence of Barrett's esophagus in the general population: an endoscopic study. Gastroenterology. 2005 Dec;129(6):1825-31.
17. Gerson LB, Shetler K, Triadafilopoulos G. Prevalence of Barrett's esophagus in asymptomatic individuals. Gastroenterology. 2002;123(2):461-7.

18. Rex DK, Cummings OW, Shaw M, Cumings MD, Wong RKH, Vasudeva RS, et al. Screening for Barrett's esophagus in colonoscopy patients with and without heartburn. Gastroenterology. 2003;125(6):1670-7.
19. Ward EM, Wolfsen HC, Achem SR, Loeb DS, Krishna M, Hemminger LL, et al. Barrett's Esophagus Is Common in Older Men and Women Undergoing Screening Colonoscopy Regardless of Reflux Symptoms. The American Journal of Gastroenterology. 2006;101(1):12-7.
20. Sikkema M, de Jonge PJ, Steyerberg EW, Kuipers EJ. Risk of Esophageal Adenocarcinoma and Mortality in Patients With Barrett's Esophagus: A Systematic Review and Meta-Analysis. Clin Gastroenterol Hepatol. 2009 Oct 19.
21. Galipeau PC, Li X, Blount PL, Maley CC, Sanchez CA, Odze RD, et al. NSAIDs modulate CDKN2A, TP53, and DNA content risk for progression to esophageal adenocarcinoma. PLoS Med. 2007 Feb;4(2):e67.
22. Chao DL, Sanchez CA, Galipeau PC, Blount PL, Paulson TG, Cowan DS, et al. Cell Proliferation, Cell Cycle Abnormalities, and Cancer Outcome in Patients with Barrett's Esophagus: A Long-term Prospective Study. Clin Cancer Res. 2008 November 1, 2008;14(21):6988-95.
23. Jin Z, Cheng Y, Gu W, Zheng Y, Sato F, Mori Y, et al. A Multicenter, Double-Blinded Validation Study of Methylation Biomarkers for Progression Prediction in Barrett's Esophagus. Cancer Res. 2009 May 15, 2009;69(10):4112-5.
24. Schulmann K, Sterian A, Berki A, Yin J, Sato F, Xu Y, et al. Inactivation of p16, RUNX3, and HPP1 occurs early in Barrett's-associated neoplastic progression and predicts progression risk. Oncogene. 2005 Jun 9;24(25):4138-48.
25. Dunn JM, Rabinovitch PS, Oukrif D, Bown SG, Novelli MR, Lovat LB. Comparison of image cytometry and flow cytometry for detection of DNA ploidy abnormalities in Barrett's oesophagus. Biochemical Society Transactions. 2009;In press.
26. Fang M, Lew E, Klein M, Sebo T, Su Y, Goyal R. DNA abnormalities as marker of risk for progression of Barrett's esophagus to adenocarcinoma: image cytometric DNA analysis in formalin-fixed tissues. Am J Gastroenterol. 2004 Oct;99(10):1887-94.
27. Murray L, Sedo A, Scott M, McManus D, Sloan JM, Hardie LJ, et al. TP53 and progression from Barrett's metaplasia to oesophageal adenocarcinoma in a UK population cohort. Gut. 2006 Oct;55(10):1390-7.
28. Lao-Sirieix P, Lovat L, Fitzgerald RC. Cyclin A immunocytology as a risk stratification tool for Barrett's esophagus surveillance. Clin Cancer Res. 2007 Jan 15;13(2 Pt 1):659-65.
29. R. Jones KCIW. The Gastro-oesophageal Reflux Disease Impact Scale: a patient management tool for primary care. Alimentary Pharmacology & Therapeutics. 2007;25(12):1451-9.
