# Supplementary material for: Evaluation of a Minimally Invasive Cell Sampling Device Coupled with Assessment of Trefoil Factor 3 Expression for Diagnosing Barrett's Esophagus: A Multi-Center Case–Control Study
Source: PLoS Med. 2015 Jan 29;12(1):e1001780. doi: 10.1371/journal.pmed.1001780 (PMC4310596; doi:10.1371/journal.pmed.1001780)
Supplement: S1 Table — (DOCX) [file pmed.1001780.s004.docx]

**Table S1.** Number of controls and cases recruited by each center.

| **Centre** | **Location** | **Total n#** | **N# Controls** | **N# Cases** |
| --- | --- | --- | --- | --- |
| Addenbrooke’s Hospital | Cambridge | 445 | 280 | 165 |
| University College London Hospital | London | 226 | 61 | 165 |
| Royal Victoria Hospital | Newcastle | 178 | 57 | 121 |
| Nottingham Queen’s Medical Centre | Nottingham | 123 | 44 | 79 |
| Queen Alexandra | Portsmouth | 32 | 4 | 28 |
| North Tyneside General Hospital | North Shields | 22 | 9 | 13 |
| South Tyneside NHS Foundation Trust | South Tyneside | 21 | 0 | 21 |
| University Hospital of North Tees | Stockton-On-Tees | 18 | 2 | 16 |
| University Hospital of North Durham | County Durham and Darlington | 17 | 1 | 16 |
| St Mark’s Hospital | London | 16 | 5 | 11 |
| Queen Elizabeth II Hospital | Welwyn Garden | 12 | 0 | 12 |
