# Supplementary material for: Evaluation of a Minimally Invasive Cell Sampling Device Coupled with Assessment of Trefoil Factor 3 Expression for Diagnosing Barrett's Esophagus: A Multi-Center Case–Control Study
Source: PLoS Med. 2015 Jan 29;12(1):e1001780. doi: 10.1371/journal.pmed.1001780 (PMC4310596; doi:10.1371/journal.pmed.1001780)
Supplement: S3 Table — (DOCX) [file pmed.1001780.s006.docx]

**Table S3:** Sensitivity and Specificity by Centre

|  | **Specificity** | **95%CI** | **Controls** | **Sensitivity** | **95%CI** | **Cases** | **Total** |
| --- | --- | --- | --- | --- | --- | --- | --- |
| Cambridge | 0.93 | 0.887-0.954 | 268 | 0.88 | 0.813-0.924 | 153 | 421 |
| Newcastle | 0.89 | 0.77-0.957 | 53 | 0.89 | 0.817-0.939 | 117 | 170 |
| Nottingham | 0.93 | 0.801-0.985 | 41 | 0.79 | 0.674-0.881 | 67 | 108 |
| others | 0.82 | 0.597-0.948 | 22 | 0.73 | 0.64-0.811 | 112 | 134 |
| UCL | 0.98 | 0.912-1 | 61 | 0.70 | 0.62-0.773 | 147 | 208 |
| **Overall** | **0.92** | **0.895-0.947** | **445** | **0.80** | **0.764-0.83** | **596** | **1041** |
